# Hierarchical taxonomy of psychopathology and personalized mental health treatment selection

**DOI:** 10.3389/fpsyt.2025.1597879

**Published:** 2025-10-29

**Authors:** Evangelia Argyriou, Christiana Prestigiacomo, Douglas B. Samuel, Jesse C. Stewart, Wei Wu, Melissa A. Cyders

**Affiliations:** ^1^ Department of Psychiatry, Indiana University School of Medicine, Indianapolis, IN, United States; ^2^ Department of Psychology, Indiana University Indianapolis, Indianapolis, IN, United States; ^3^ Department of Psychological Sciences, Purdue University, West Lafayette, IN, United States

**Keywords:** HiTOP, precision mental health, treatment response, CBT, internalizing

## Abstract

Accumulated research has shown considerable heterogeneity in mental health treatment response. Precision mental health approaches aim to leverage this heterogeneity to tailor treatment selection to individual needs. The goals of this manuscript are to 1) present theoretical rationale for the potential usefulness of the Hierarchical Taxonomy of Psychopathology (HiTOP) to optimize treatment selection and 2) conduct a scoping review of the role of individual psychopathology components that map onto HiTOP on differential psychotherapy response, both as a proof-of-concept analysis, as well as to identify gaps and concrete recommendations for future application. We focus our review on treatment for internalizing disorders as a candidate class of disorders, and on Cognitive-Behavioral Therapies given their empirical support for this disorder class. Overall, the reviewed literature provides preliminary evidence about the potential usefulness of HiTOP dimensions of differing levels of specificity for personalized treatment selection that can guide future research. Gaps and limitations were identified, including limited research in several HiTOP domains, strict inclusion/exclusion criteria shrinking individual heterogeneity, large variability in HiTOP dimension measurement, risk of Type I and Type II error, and other methodological limitations for assessing personalized treatment response. The translation of this research to clinical decision making has a long way to go. Nonetheless, we view the application of HiTOP-relevant dimensions to personalized mental health approaches as a viable and exciting direction that offers many avenues for research for the improvement of patient outcomes.

## 1 Introduction

Consider a 35-year-old client presenting to an outpatient clinical setting with complaints of increasingly depressed mood, anxiety, and fatigue. Various evidence-based treatments, including second- and third-wave cognitive behavioral therapies (CBTs), interpersonal psychotherapy (IPT), and psychiatric overseeing for medication, are available in this setting. How should clinicians build the treatment plan based on the available options? The gold standard is to utilize the diagnostic label (e.g., based on the Diagnostic and Statistical Manual of Mental Disorders 5th ed.; DSM–5; 1) to guide clinical decision making about treatment selection. However, decades of research show that, on average, there is little systematic difference among validated mental health treatments for a given diagnosis (e.g., 2–4). Some have argued that this means all treatments are equally effective, lack specificity with respect to treatment outcome, and exert an effect through common factors shared among psychotherapies (5–7). If all treatments are equally effective for everyone, treatment selection would be a straightforward process mainly based on clinician training and skills and on client preferences. However, research on behavioral and pharmacological interventions often yields small to medium effects (8–11). One reason for the small to moderate magnitude of treatment effects may be that the effects correspond to the average individual and, thus, do not consider individual variability in treatment response (12). Thus, treatments may be equally effective on average, but not for each person.

Individual clients respond differently to a given treatment (13–16). For example, within a population, some experience substantial benefits, others experience no benefits, and others experience a worsening of symptoms in response to treatment. Therefore, the average treatment effect, which is the main outcome of interest in most clinical trials (17), is too crude to describe the suitability and superiority of an intervention for a given individual. The average treatment effect provides little information for practitioners to make optimal personalized treatment choices. To address this issue, precision medicine (or precision mental health, as applied in mental health research) approaches have started to gain ground, aiming to leverage individual heterogeneity affecting treatment response in order to tailor treatment selection to individual needs and to maximize treatment benefits for individuals (12, 18). In this approach, the question is not which treatment is best, but rather, which treatment is best for which individuals. This field is growing rapidly, and methodology to address precision mental health questions is an area of open investigation (19). However, to realize the promise of precision mental health approaches, one important question is central: Which variables account for the heterogeneity in treatment response and should be considered for personalized treatment selection? In this paper, we present a theoretical rationale concerning the utility of individual psychopathology dimensions organized by the Hierarchical Taxonomy of Psychopathology (HiTOP; see 20) framework to capture relevant heterogeneity in treatment response. HiTOP proposes an empirically driven dimensional hierarchical structure of psychopathology with dimensions of increasing levels of generality (from groupings of symptoms into groupings of highly related syndromes, i.e., spectra) that are theorized to reflect underlying mechanisms of clinical presentations more accurately and more reliably.

Thus, the goal of this manuscript is two-fold:

1. To present a theoretical justification about the potential usefulness of the HiTOP framework for personalized treatment selection. We seek to build the rationale for the hypothesis that using the HiTOP framework and measuring different specificity levels of psychopathology components across different spectra simultaneously will optimize psychotherapy selection.
2. To conduct a scoping review on the role of individual psychopathology components that map onto HiTOP on differential treatment response, both as a proof-of-concept analysis, as well as to identify gaps and concrete recommendations for future application. Given the recency of the proposed HiTOP framework, there are few studies directly testing this hypothesis. Thus, we present evidence from a scoping review of studies that examined one or more HiTOP-relevant moderators of differential response to a set of psychotherapies.

### 1.1 From evidence-based treatment to precision mental health

“What treatment, by whom, is most effective for this individual with that specific problem, and under which set of circumstances?” Gordon Paul (21) posed that question over 50 years ago, but the “*what* works for *whom*” question continues to be as timely as ever and may be finally within our reach, given recent advancements in research design, technology, and statistical methodology allowing empirical examination of this seemingly simple, yet complex question. Implicit in this question is the recognition that no single treatment is best for everyone and that a variety of individual characteristics dictate the type of treatment that will be more beneficial for a given individual at a given time.

Initial attempts to answer this question were rooted in evidence-based practice and the identification of empirically supported treatments, incorporating evidence from randomized clinical trials that test the efficacy of a given treatment for a specific DSM-defined disorder compared to other control or treatment conditions (22). The primary focus of such trials is the identification of the best treatment for a given disorder by looking at the average treatment effect, with any heterogeneity in treatment response that could come from client characteristics being viewed as a nuisance or error variance (19). Therefore, although the goal in evidence-based practice is to take such characteristics into account in clinical decision making, underlying evidence-based research gives limited direct empirical support for how to do this because individual differences are disregarded and averaged. Thus, clinical decisions have often been left to clinical judgment, patient preference, and to practical considerations, such as availability and cost of a treatment (23). Precision mental health aims to individualize the healthcare process to the uniquely evolving needs of each client through *leveraging* individual heterogeneity (i.e., variability in an array of characteristics that make a person unique) (12), rather than treating it as nuisance variance.

### 1.2 Personalized mental health treatment selection

Precision medicine and precision mental health research encompass various areas of open investigation that can ultimately contribute to the individualization of health care: risk factors, prognostic factors, and treatment selection. The first two areas focus on the identification of biomarkers and other factors shared within subpopulations of individuals that make them differentially susceptible to a given medical or mental health condition or to a specific prognosis for that condition (24, 25). For example, sequencing of lung adenocarcinomas led to the identification of more than 15 different gene mutations (e.g., KRAS or EGFR mutation) related to lung cancer (26). Research in this area enables personalized diagnosis and can be particularly important for the development of novel treatments addressing specific risk and/or prognostic factors. In contrast, personalized treatment selection involves considering available treatment options and optimizing their allocation to the individuals that will be most benefited based on a variety of individual characteristics (12, 24).

The concept of personalized treatment selection has been loosely defined in many ways to directly or indirectly improve treatment selection. For the purposes of this paper, we define personalized treatment selection as a methodological approach that directly estimates a data-driven set of decision rules, resulting in a recommendation of the optimal treatment choice (from a set of treatment options) for an individual that maximizes a clinical outcome. These decision rules (also known as personalized treatment rules) can involve a single decision point (e.g., prescribe CBT vs. Acceptance and Commitment Therapy [ACT]) or multiple decision points (i.e., sequence of rules based not only on the baseline values of individual characteristics but also on their change over time along with ongoing performance of the interventions (also called dynamic treatment regimes or adaptive interventions) (19, 27, 28)). Available treatment options that can be considered range from different types of behavioral and pharmacological interventions, various modes of delivery (e.g., individual vs. group format, in person vs. online), combinations of intervention (e.g., motivational interviewing + CBT + anti-depressant medication vs. motivational interviewing + CBT vs. CBT only), or different intervention timelines (e.g., start with behavioral activation and if effective move to cognitive restructuring vs. start with cognitive restructuring).

Personalized treatment rules are estimated based on characteristics that differentially influence treatment response and indicate which treatment is estimated to be best for whom at a given decision point (see 29, 30 for examples of personalized treatment rule estimation for treatment prescription). Such characteristics are often referred to as treatment moderators. Moderators can be either predictive (e.g., Treatment A is always better than Treatment B, but the difference is greater at higher levels of anxiety; we would still prescribe Treatment A regardless of anxiety) or prescriptive (e.g., at high levels of anxiety Treatment A is better than Treatment B and at low levels of anxiety Treatment B is better than Treatment A; at high levels of anxiety we would prescribe Treatment A whereas at low levels of anxiety we would prescribe Treatment B). This distinction is important for personalized treatment selection because prescriptive moderators reflect differential superiority of a treatment depending on levels of the moderator, whereas predictive moderators do not. Prescriptive moderators are also known as treatment prescriptive variables or tailoring variables (27).

Treatment moderators have traditionally been studied by examining the interaction effect between the putative moderator and treatment condition in a standard regression analysis (23). A significant interaction effect is often followed by *post hoc* probing, which examines the treatment effect at different levels of the moderator (e.g., mean, at 1 standard deviation (SD) above and below the mean for a continuous moderator or for each group of a categorical moderator) (31). Although this approach has advantages, such as familiarity and ease of use, it only indirectly gives information about optimal treatment selection. In other words, it does not produce a specific decision rule that can be applied for a new client seeking treatment with specific demographics and/or scores on their assessment measures (e.g., Beck Depression Inventory = 25 and Beck Anxiety Inventory = 5, and Alcohol Use Disorders Identification Test = 30) and their individual demographics. Other more sophisticated approaches based on prescriptive algorithms that generate data-driven rules for optimal treatment recommendations, referred to in the rest of the review as personalized treatment rules, have started to appear in the literature (for a review of personalized treatment selection methods currently used in mental health research, see 12; 23). Such approaches enable inserting the new client’s scores in the algorithm, with output being the recommendation of the treatment from a set of previously studied treatments that would benefit them the most. Research has shown that treatment assignment based on such rules outperforms that of clinician judgment, which is naturally influenced by multiple types of bias (e.g., confirmation bias; 32). Although there is still a lot to be learned before the dissemination in practice, this evidence shows the promise of such approaches for the individualization of clinical decision making.

Optimal personalized treatment selection is a sophisticated endeavor, and, as such, optimal study designs, rigorous statistical method development, and inference are active areas of investigation (19, 33, 34). The same holds true for the variables that would be useful to tailor treatment selection. Such prescriptive variables can vary from demographic characteristics, genetic, epigenetic, neurobiological, and cognitive variables, environmental influences, social context, cultural background, family history, as well as psychopathology presentation.

### 1.3 Hierarchical taxonomy of psychopathology and personalized treatment response

Psychopathology presentation is formally captured in diagnostic systems. Diagnostic systems were developed to facilitate research on the etiology of mental illness and the development of interventions that effectively address underlying mechanisms of mental illness for groups of individuals that share similar characteristics. Thus, a reliable, valid, and clinically useful diagnostic system is a cornerstone of treatment planning and clinical decision making (35). Although traditional diagnostic systems may be useful for testing overall effectiveness of treatment, they fall short in facilitating personalized treatment selection because they do not sufficiently capture the inherent heterogeneity of psychopathology. Instead, they yield a diagnostic categorical label (with some specifiers) assigned to multiple patients with potentially very different presentations. For example, a study found that 1,500 patients with DSM-IV major depressive disorder (MDD) diagnosis met symptom criteria in 170 different ways (36). Two people sharing only one clinical symptom could be given the same diagnosis of MDD - meaning that there is very little overlap in their underlying characteristics. Additionally, traditional diagnostic systems also result in large overlap between diagnoses. For example, MDD and generalized anxiety disorder (GAD) diagnoses share four overlapping clinical criteria, creating more similarity than differences between these diagnoses. This is also reflected in the high rates of comorbidity among these disorders 45.7%–75.0% (37–39). These limitations make categorical diagnoses less useful in capturing individual heterogeneity that may uniquely explain variability in treatment response.

Alternative frameworks of psychopathology, such as HiTOP or the Research Domain Criteria (RDoC), seek to address the limitations of traditional diagnostic systems by incorporating dimensional data-driven constructs that are theorized to reflect underlying mechanisms of clinical presentations more accurately (35; 40–42). RDoC focuses on domains of psychological functioning (i.e., negative valence, positive valence, cognitive systems, systems for social process, arousal/regulatory systems, sensorimotor systems and neural circuits that underlie these dimensions (measured by behavioral performance, self-reports, physiology; 40, 41). Although the RDoC system holds promise for uncovering the biological basis of psychopathology, it does not cover clinical phenomena with sufficient detail, and research attempting to map biology on to clinical phenotypes is in its infancy, leading to some concluding that it has limited usefulness in clinical practice, at least for the time being (20). Additionally, although the biological information relevant to RDoC may be highly important for predicting and explaining personalized treatment response, it is expensive to assess and rarely accessible to clinical practitioners offering behavioral interventions. Thus, there is a clear need to investigate behavioral characteristics that potentially have biological underpinnings and can be easily and routinely assessed in clinical practice as potential determinants of heterogeneous treatment response.

The HiTOP system (Figure D.1) is a hierarchical alternative to traditional diagnostic systems that conceptualizes psychopathology as a set of homogeneous dimensions organized into increasingly broad, transdiagnostic dimensions that account for comorbidities (20, 35, 43–45). It is based on a data-driven re-organization of DSM symptoms and, as such, maximizes homogeneity within each dimension and heterogeneity across different dimensions. The high degree of granularity allows more effective capture of the inherent heterogeneity within psychopathology. The HiTOP framework enables the investigation of individual psychopathology profiles of differing levels of specificity (20, 44), from fine grain symptom components and traits (e.g., insomnia, anxiousness, emotional lability) to general higher order transdiagnostic dimensions (e.g., the spectra and superspectrum). Specifically, this system enables the measurement of a detailed profile of 1) higher-order general predispositions to psychopathology that are thought to reflect common genetic/biological underpinnings and 2) lower-order trait and symptom components, the composition of which may differ considerably across individuals. Different levels of dimension specificity may partly explain the significant heterogeneity in treatment response. This framework, in contrast to categorical diagnoses, considers differences among individuals who share subclinical or clinical levels of psychopathology. As such, each dimension is relevant to all individuals. HiTOP reorganizes DSM symptoms into hierarchical and homogeneous dimensions; thus, the contents of this model, the granules, are not inherently novel. The novelty and usefulness of this framework for personalization has to do with the organization into a hierarchical structure and the comprehensiveness it can provide in assessing symptom and trait profiles in a systematic way, providing simultaneously a zoomed out and zoomed in picture of clinical presentation that can be used as input to estimate individualized clinical decision rules.

### 1.4 HiTOP dimensions as potential prescriptive factors for personalized treatment selection

HiTOP dimensions of differing levels of specificity show promise to differentially predict treatment response (35, 46) and, thus, inform personalized clinical decisions. This makes HiTOP a prime candidate framework for personalized diagnosis and treatment. For instance, a client whose elevated depression is particularly driven by lethargy and irritability may benefit more from a treatment incorporating behavioral activation than from one focusing on problematic interpersonal relationships. The opposite may be true for someone with higher detachment level, who may benefit more from a treatment focusing on improving interpersonal functioning rather than one focused on behavioral activation. Additionally, a general disposition towards antagonistic externalizing is linked with relationship dissatisfaction and conflict (47), which may lead to interference with therapeutic alliance and the clinician’s efforts to build rapport. Thus, a client with this disposition might benefit from the addition of motivational interviewing to improve treatment adherence (48). Thus, understanding one’s individual characteristics from a HiTOP perspective can inform numerous clinical decisions and, in theory, may lead to more optimal treatment selection.

Traditionally, heterogeneity in psychopathology has been considered an inconvenience that, at best, makes the work of researchers and clinicians more difficult. However, if evidence is provided that psychopathology heterogeneity can improve, instead of hinder, clinical decision making, it will encourage researchers to develop more reliable approaches for personalized treatment selection, taking into account individual psychopathology profiles along with other important baseline characteristics. Insights from modern precision medicine approaches can be used to provide parsimonious personalized treatment rules to tailor interventions to the individual’s unique profile, which can be used readily by the clinician (see Figure E.1 for an example of what such an algorithm based on HiTOP dimensions could look like in practice). To approach this goal, research needs to directly study whether and, if so how, HiTOP dimensions could be combined to develop personalized treatment rules that improve treatment outcomes. However, such research is in its infancy.

## 2 Scoping review of HiTOP-related moderators of differential treatment response

The goals of this scoping review are to 1) provide a proof-of-concept analysis for the usefulness of HiTOP dimensions in predicting differential treatment response, 2) identify gaps and limitations of existing research that prevent applying HiTOP to personalized mental health treatment selection, and 3) formulate directions for future research in personalized mental health treatment selection.

As a starting point, we focused our review on two areas: First, a focus was placed on treatment for disorders fitting into the internalizing spectrum (e.g., MDD, GAD, eating disorders) as a candidate class of disorders to test personalized treatment response based on HiTOP moderators, because there is more research available with a variety of HiTOP moderators tested that would allow us to examine our question. The internalizing spectrum consists of symptoms/traits and disorders that share common features related to emotional dysfunction and/or behavioral avoidance (49). Second, a focus was placed on CBTs as the primary treatment of interest because CBTs are well validated and widely used psychotherapy approaches proven to be effective for internalizing disorders (e.g., 50). However, there is still variability in response to CBTs for this disorder class, which means that identification of viable prescriptive factors would improve optimal assignment to CBTs versus other psychotherapies (or among CBT variations) for more effective treatment.

### 2.1 Search terms and strategy

Articles were identified by searching PsycINFO and PubMed databases. Search terms included were HiTOP-related dimensions, psychotherapy-related terms, and moderator analysis and precision medicine/mental health-related terms (a full list of the search terms can be found in **Appendix A**. Only empirical peer-reviewed studies in English were included in the review. Treatment response to CBT versus other behavioral treatment or across different CBTs was explored. Third wave CBTs with moderate/strong evidence of efficacy/effectiveness (51), including Acceptance and Commitment Therapy (ACT), Dialectical Behavior Therapy (DBT), Behavioral Activation, and Mindfulness-based Cognitive Therapy (MBCT) were also included in the review. Thus, to be included, studies had to examine the effect of CBT 1) compared to at least one active behavioral treatment (this could be a different variation of CBT, treatment as usual based on behavioral interventions, other behavioral treatment) on clinical outcomes and 2) for an internalizing disorder as defined by HiTOP (e.g., mood disorders, anxiety disorders, eating disorders, sexual dysfunctions). Studies examining prevention interventions were excluded. To be included, studies had to quantitatively assess clinical outcomes directly relevant to mental health improvement both pre- and post-treatment and/or at follow-up time points.

Clinical outcomes included, but were not limited to, reduction in symptom severity, improvement in functioning, remission, and recovery. Studies examining clinical outcomes not directly linked to mental health improvement, such as treatment readmission, dropout, and treatment length, were excluded. In addition, studies had to examine at least one psychopathology dimension (measured continuously) of any level of specificity – superspectrum, spectrum, subfactor, syndrome, or symptom component/trait – as a treatment moderator measured at baseline, prior to treatment initiation. The HiTOP consortium recommends the use of a set of HiTOP-friendly measures (52). However, due to limited research with these particular measures in the context of our review we expanded our inclusion to any dimensional measures assessing the above constructs. Dimensions were selected based on terms used by the HiTOP consortium (20, 43). These terms were supplemented by measures suggested by the HiTOP consortium and the HiTOP clinical network to capture the proposed dimensions (52). Symptom component dimensions from disorder-specific symptom measures were also included (e.g., focusing on eating pathology), as they assess narrower aspects of nosology that have not yet been officially included in the HiTOP hierarchy but are consistent with the framework (20). Normative personality traits of the Five Factor Model, the extremes of which have been conceptualized to correspond to pathological personality traits, negative affectivity, detachment, antagonism, and disinhibition (53), were also included in the review as HiTOP-relevant moderators (spectrum-level specificity). These normative, and their corresponding pathological, personality traits were classified as spectrum-level moderators. HiTOP-relevant moderators were categorized to the broader spectra and the specificity levels based on the proposed HiTOP model.

HiTOP-relevant moderators were assigned to a broader spectrum domain (or General Psychopathology) – i.e., Internalizing, Disinhibited Externalizing, Antagonistic Externalizing, or Detachment – and a specificity level – i.e., superspectrum, spectrum, subfactor, syndrome, or component/trait. Given that the HiTOP system is a work in progress, the classification of some components of the model to their corresponding spectra was more certain than others based on existing research. For this reason, the certainty of fit of each moderator to a given spectrum was labeled as 1 = high vs. 0 = low certainty. For example, research has consistently classified depression and anxiety under the internalizing spectrum. Thus, such components were assigned a value of high certainty of fit to their assigned spectrum. On the other hand, there is mixed evidence about the place of bipolar disorder, eating disorders, or obsessive-compulsive disorder (OCD) and related components in the model, as they have been found to load onto different spectra across studies (54–56). Such components were assigned a value of low certainty of fit to their assigned spectrum.

Each study was assigned a rating of low, high, or unclear methodological risk of bias from two sources, i.e., selection bias and detection bias (as indicated by whether studies used random sequence generation, allocation concealment, and blinding of outcome assessment), as study quality indicators based on the Cochrane Handbook (57) and the guidelines of the Cochrane Consumers and Communication Review Group (58). Variables for sample size, method type, multiple comparison correction, and examination of nonlinear moderating effects were also created. This was done to qualitatively assess the statistical quality of the studies, i.e., appropriateness of the analysis, Type I and Type II error in the findings, and possibility of misspecification of the models (assuming linearity in potentially nonlinear moderating effects). Descriptive statistics were computed for mean sample size, age, sex (% female), and race (% non-white). All identified articles were coded by the first author, and 20% of these were coded by an independent coder, the second author, to calculate inter-rater reliability which was 95.7%.

### 2.2 Scoping review results

Included studies (59–133) and general findings are presented in Table B.1 (an inclusion flowchart is shown in Figure F.1). Seventy-six studies met criteria for inclusion in this scoping review. Characteristics of the included studies are presented in **Table 1**.

**Table 1:** Characteristics of included studies.

Study characteristics	Total	Significant moderation	Nonsignificant moderation
	Median (Q1, Q3)	Median (Q1, Q3)	Median (Q1, Q3)
Sample size	127 (80, 235)	126 (80, 220)	128 (99, 251)
Mean age	38.71 (35.15, 42.56)	36.80 (34.75, 40.30)	39.73 (35.72, 44.35)
% Female	66.90 (57.00, 75.36)	68.20 (49.58, 85.30)	66.20 (59.95, 72.90)
% Non-White*	21.36 (11.85, 31.05)	19.71 (11.17, 31.30)	24.00 (12.00, 30.30)
	Total	Significant moderation	
	N	N	%
Comparison treatment			
CBT	31	21	67.74
Non-CBT	45	24	53.33
Design			
Randomized trial	68	41	60.29
Non-randomized trial	3	1	33.33
Retrospective based on electronic health records	3	1	33.33
Mix of randomized and non-randomized datasets	1	1	100.00
Naturalistic study	1	1	100.00
Risk of bias			
Random sequence generation			
Low	44	25	56.82
High	9	5	55.56
Unclear	23	15	65.22
Allocation concealment			
Low	27	14	51.85
High	13	9	69.23
Unclear	36	22	61.11
Blinding outcome assessment			
Low	37	23	62.16
High	28	16	57.14
Unclear	11	6	54.55

* Missing in 42 studies.

HiTOP-relevant dimensions have been evaluated as moderators of differential response to treatment for internalizing disorders classified into four disorder classes mapping onto the HiTOP model: distress disorders, eating disorders, fear disorders, and a general internalizing class combining one or more internalizing disorders. Overall, all HiTOP specificity levels and spectra have been evaluated as moderators of differential treatment response. However, most areas were not well-researched. Almost half of the included studies examined treatment for distress-related disorders (*k* = 36), and the vast majority of these studies examined depression (*k* = 27). Treatment for eating (*k* = 14) and fear (*k* = 16) disorders was less well represented in the review. Although there were many distinct HiTOP-related moderators examined in the reviewed studies, these were primarily relevant to the Internalizing spectrum (e.g., syndrome level anxiety and depression, and a variety of component-trait level moderators such as avoidance, hostility, and affective lability). Other spectra –Somatoform, Thought Disorder, Antagonistic and Disinhibited Externalizing, and Detachment spectra – were much less explored. Additionally, lower specificity moderators (i.e., higher-order factors) at the superspectrum, spectrum, and subfactor-level were less well studied than HiTOP dimensions of higher specificity (i.e., lower-order factors).

Overall, 59.2% of the studies found at least one significant HiTOP-relevant moderator of differential treatment response (see **Table 2**). Table C.1 shows all the HiTOP-relevant moderators examined in the included studies across disorder classes and whether there is evidence of significance at least once. The heatmaps in Figure G.1 depict the percentage of significant moderating effects within spectra and across different specificity levels in the four disorder classes. Of the studies examining distress disorder (*k* = 36), fear disorders (*k* = 16), and more than one internalizing disorder (*k* = 10), approximately half found significant HiTOP-relevant moderators. Studies of eating disorder (*k* = 14) found a higher proportion of at least one significant moderator (71.4%). Although there was variability in the proportion of significant effects for different levels of specificity, most consistent moderators were found at the syndrome (44.68%) and component/trait level (49.32%). Studies examining internalizing moderators were the most likely to find significant moderating results across spectra (47.76%).

**Table 2:** Number and percentage of studies that found at least one HiTOP-relevant significant moderator.

HiTOP dimensions	Total studies			Distress disorders			Eating disorders			Fear disorders			One or more internalizing disorders		
Specificity	Sig	%	Total	Sig	%	Total	Sig	%	Total	Sig	%	Total	Sig	%	Total
Superspectrum	3	25.00	12	3	37.50	8	0	0.00	1	0	0.00	1	0	0.00	2
Spectrum	4	28.57	14	4	50.00	8	0	0.00	1	0	0.00	2	0	0.00	3
Subfactor	3	42.86	7	0	0.00	1	3	50.00	6						
Syndrome	21	44.68	47	9	37.50	24	2	33.33	6	7	70.00	10	3	42.86	7
Component/trait	26	48.15	54	12	46.15	26	8	72.73	11	2	20.00	10	4	57.14	7
Spectra															
General psychopathology	3	25.00	12	3	37.50	8	0	0.00	1	0	0.00	1	0	0.00	2
Somatoform	1	25.00	4	1	33.33	3							0	0.00	1
Internalizing	36	49.32	73	14	40.00	35	10	71.43	14	8	53.33	15	4	44.44	9
Thought Disorder	4	40.00	10	3	37.50	8							1	50.00	2
Disinhibited EXT	5	31.25	16	2	22.22	9	1	33.33	3	1	50.00	2	1	50.00	2
Antagonistic EXT	4	30.77	13	3	30.00	10	1	100.00	1				0	0.00	2
Detachment	5	26.32	19	4	33.33	12	1	33.33	3	0	0.00	3	0	0.00	1
Total	45	59.21	76	21	58.33	36	10	71.43	14	9	56.25	16	5	50.00	10

Sig = Number of studies finding at least one significant moderator, EXT = Externalizing. Shaded cells show results for moderators assessed in at least 3 studies. Cell color corresponds to the strength of evidence as quantified by percentage size.

Importantly, there was not a consistent pattern or direction of moderating effects across therapies and disorders. This varied across spectra and disorder classes. For example, in studies on distress disorders, spectrum-level moderators were found significant only at the Detachment (e.g., 59, 60) and Thought Disorder level (e.g., 61). Within the Detachment spectrum, component/trait-level moderators seemed to be less viable compared to spectrum and syndrome level (e.g., 59, 60, 62, 63). Additionally, component/trait level moderators of spectra outside the Internalizing may be more viable compared to internalizing moderators at that level, although few studies were conducted to make definitive conclusions. Within Internalizing, syndrome-level dimensions may be viable to moderate differential treatment response for distress disorders (e.g., 59, 60, 64, 100). Within Detachment, spectrum-level dimensions may be more viable (e.g., 60). This may suggest that different components of the HiTOP model play a unique role on treatment response that can be leveraged for personalized treatment selection. However, given that the amount of research is skewed towards Internalizing moderators, a fair comparison of evidence among spectra and disorder classes cannot be made.

### 2.3 Scoping review discussion

Overall, the current scoping review found some preliminary signal for the usefulness of HiTOP-dimensions in personalized mental health treatment selection. Importantly, we purposefully restricted our review to internalizing disorders and CBTs, as literature in this domain examining HiTOP-relevant moderators is more robust than for other disorders and treatments. Even for this set of disorders and treatments, the literature was limited; we discuss these limitations more fully below and view them as prime opportunities to advance research into personalized treatment response and selection. Although we cannot make a firm judgment about whether HiTOP-relevant moderators are important for other disorders and treatments, these preliminary results suggest that viability to study HiTOP dimensions as predictors of differential treatment response and optimal treatment selection of CBTs for the treatment of internalizing disorders and then to continue this research for other disorders and treatments in the future.

We believe this review provides adequate proof-of-concept to further study and test HiTOP-relevant dimensions for applications to personalized treatment selection. However, exactly which factors, and how to leverage them, and for which disorders and treatments, cannot yet be determined by the existing literature. The literature reviewed suggests that the full range of the HiTOP model should be tested for personalized treatment prescription. Given heterogeneity in directions of moderation effects, it is important that a comprehensive selection of putative moderators relevant to the examined set of treatments is made. If we consider them in isolation, scores of a person in one dimension may indicate one treatment and scores on another dimension may indicate another, which would not be helpful in clinical practice (99, 100). Parsimony of the personalized treatment rules is critical because it will help clinicians personalize assessment batteries to measures that capture only characteristics relevant to treatment selection. This will improve assessment efficiency, reduce patient burden, and optimize the treatment selection process.

Based on the reviewed studies, we propose that whether a particular HiTOP dimension is useful may depend on several factors. First, usefulness may depend on the specific patient population to which the treatment is addressed (i.e., condition treated). For example, in studies on distress disorders, spectrum-level moderators were found significant only at the Detachment (e.g., 59, 60) and Thought Disorder level (e.g., 61). Second, usefulness may depend on the spectrum to which it belongs (i.e., within or outside spectrum to which the outcome is classified). For example, within the Internalizing spectrum, syndrome-level dimensions may be viable to moderate differential treatment response for distress disorders (e.g., 59, 60, 64, 100). Within the Detachment spectrum, component/trait-level moderators seemed to be less viable compared to spectrum and syndrome level (e.g., 59, 60, 62, 63). It is also worth noting that moderators that were very similar or identical to the assessed outcome (e.g., depression level at baseline as a moderator of the effect of treatment on depression score at treatment completion) were more likely to significantly moderate differential treatment response. This is reflective of the potential importance of baseline severity for treatment selection, which may be especially relevant for the selection of higher versus lower intensity treatments (e.g., 60). However, no clear pattern was observed as the moderators conceptually moved away from the assessed outcome, regardless of whether it was a component within the same spectrum or a construct from a different spectrum.

Third, usefulness of HiTOP dimensions for predicting treatment response may depend on the specificity level of the dimension and/or the specific set of treatment conditions compared and the extent to which the dimension is relevant to them and can differentiate among them. For example, component/trait level moderators of spectra outside the Internalizing spectrum may be more viable compared to internalizing moderators at that level, although few studies were available for making definite conclusions. Additionally, HiTOP dimension spectrum and specificity, as well as the condition treated may have district contributions depending on the different sets of treatments compared. The treatment conditions can be behavioral treatments of different theoretical orientation (e.g., CBT vs. interpersonal psychotherapy), high vs. low intensity (e.g., inpatient vs outpatient), or the same treatment with different component modifications designed to address a specific HiTOP dimension (e.g., standard CBT for depression vs. CBT for depression enhanced to specifically address weight and shape concerns).

For example, for higher levels on detachment-related moderators (outside spectrum moderators), standard CBT/CT may be associated with more improvement than interpersonal psychotherapy for a distress disorder; 64, 65). One possible explanation is that these patients might be more involved and benefited by a treatment that is more directive and concrete than one that focuses on interpersonal relationships. However, this dimension might not be a significant moderator of treatment when treatment conditions with similar levels of directiveness are compared (e.g., 66). On the other hand, baseline severity (within spectrum, syndrome-level moderator) may help differentiate between high intensity standard CBT and low intensity brief therapy (e.g., 60). This would be particularly important to maximize benefits in resource-limited settings where high intensity treatment cannot be administered to all treatment-seeking individuals. Finally, whether depression is driven by lassitude (within spectrum, higher specificity dimension) may differentiate between a treatment incorporating behavioral activation and a treatment that focuses on problematic interpersonal relationships but may not moderate standard CBT versus behavior therapy (both can include behavioral activation, thus irrelevant to treatments).

Limitations of the current review should also be noted. First, this review does not have immediate practical implications and was not intended to provide practical recommendations; instead, it focuses on general patterns of research and findings. This is due to 1) the lack of studies examining multiple moderators of different spectra and specificity levels simultaneously, that would make the interaction directions and effect sizes found misleading for personalized treatment selection purposes, and 2) lack of studies examining the same moderators and set of treatments to assess consistency of the findings. Second, the general pattern was evaluated based on statistical significance of examined moderations and did not meta-analyze these relationships, thus precluding any conclusions regarding the overall size or direction of these effects. Given the small sample size in many of the included clinical trials, it is possible that some non-significant moderations were due to reduced power. At the same time the examination of multiple tests of moderation could lead to Type I error. Third, we focused our review to the HiTOP framework and relevant dimensions that may affect treatment response. One criticism of this framework is that it is not inherently novel, and it still involves symptoms and syndromes similarly to the DSM, instead of focusing to underlying mechanisms. Therefore, the HiTOP relevant dimensions might not directly influence treatment response. Other systems might prove to be more appropriate to predict differential treatment response in the future. Until then, apart from the DSM, HiTOP is one of the most comprehensive systems covering the full range of psychopathology and psychopathology covariation that cannot be disregarded when deciding what is more beneficial treatment for a certain client. In addition, the longevity and replicability of the empirical basis for the HiTOP structure proves that it is fairly well established (100, 134–137). Based on this evidence, structural components of the model may reflect, at least partly, these common mechanisms that do directly impact differential treatment response. Third, this review included only studies with samples treated for an internalizing disorder. This limits the generalizability of the findings to only internalizing disorders. Finally, this review defined CBTs broadly to include a wide range of CBTs including 3^rd^ wave CBTs. We also included articles comparing different formats of CBTs. Although this was done to broaden the scope and review a variety of moderators, this makes the synthesis of the results more complex. However, our goal was not to aggregate effects across treatments but discuss what is available and whether there is signal about the moderating effect of HiTOP dimensions without trying to combine effects. We do encourage readers to bear these limitations in mind as they evaluate the presented results.

## 3 Discussion

### 3.1 Critical analysis of the literature reviewed

Several methodological limitations in the literature were identified in the literature that threaten the validity of conclusions, hinder progress in HiTOP-relevant research, and limit application to personalized treatment selection.

#### 3.1.1 Strict eligibility criteria shrinking individual heterogeneity

Generally, although a large number of distinct HiTOP-related moderators were examined in the reviewed studies, they were primarily relevant to the internalizing spectrum, resulting in restricted variability and possibly non-significant moderation effects. One possible reason is the often strict exclusion criteria that limit researchers’ ability to examine them comprehensively, if at all. In most of the included studies, individuals with severe psychopathology (e.g., psychotic disorder) or substance use disorders were excluded. For example, in a study where Thought Disorder dimensions were examined as potential moderators, presence of psychotic symptoms was one of the exclusion criteria for participation (59). Therefore, in these cases, the variability in the putative moderators and treatment response is likely reduced. Consequently, non-significant moderating effects found are not surprising. Low heterogeneity in patient characteristics minimizes the likelihood of finding useful prescriptive factors for personalized treatment selection. In other words, in most clinical trials where the goal is to assess average treatment effects, strict exclusion criteria are set to reduce variability in treatment response. Although this may be useful in finding one-size-fits-all approaches, it does not serve the purpose of personalized treatment selection that seeks to leverage individual heterogeneity to optimize treatment outcomes rather than treating it as nuisance variance.

#### 3.1.2 HiTOP dimension measurement

An important limitation that should be addressed in future research is the measurement of the HiTOP-relevant dimensions. In existing research, these dimensions were assessed with a multitude of different measures and were frequently constrained to measures related to the primary diagnosis of the sample (corresponding to baseline severity). Thus, the full range of psychopathology was rarely assessed comprehensively and simultaneously within a study. Relatedly, many of the symptom-based measures used were at the syndrome-level reflecting the dominance of the categorical systems (DSM/International Classification of Diseases [ICD]) in this research. This is in part due to the lack of awareness of the HiTOP model and in part due to the lack of a comprehensive and validated HiTOP measure that would enable consistency of measurement of HiTOP constructs across studies. Additionally, measures that are supposed to measure the same constructs across studies, even widely studied dimensions, such as depression or anxiety, varied significantly in content (e.g., placing different emphasis to emotional, cognitive, physical or behavioral symptoms of depression or anxiety) (138).

#### 3.1.3 Risk of type I and type II error and validity of conclusions

A given moderator was rarely examined in multiple studies with the same primary diagnosis and treatment conditions, preventing examination of reproducibility of the identified moderating effects, or lack thereof. In the few studies that examined the same moderators, moderating effect findings were often not replicated. This could be due several reasons. One is that the examined moderators were evaluated in primarily small clinical trials that most likely were not adequately powered for moderation analysis (Median N = 126, IQR = 150). Thus, null moderating effects could be a result of Type II error. The other is that many of these studies were secondary analysis of clinical trials (e.g., 59, 62) or exploratory in nature; thus, many moderating effects were often tested in separate models without *a priori* hypotheses (e.g., 67). As it is well known, multiple tests would have led to Type I error inflation. The presence of Type I and Type II errors may contribute to the inconsistent results across studies.

#### 3.1.4 Statistical approach limitations for assessing personalized treatment response

The statistical methods used varied considerably, and some were more appropriate to assess personalized response than others. For most of the reviewed studies, the goal was to address the question of what treatment works best for whom to eventually enable optimal personalized treatment selection based on baseline characteristics including (but not exclusively) different psychopathology dimensions. However, most of these studies used unrealistically simplistic models to represent complex psychopathology phenomena. For example, these studies assessed treatment moderators using standard linear regression analysis (or multilevel modeling) with only two-way interaction effects. These models are often misspecified, and do not account for non-linear effects (with few exceptions, e.g., 68; 69). However, the associations of complex psychopathology phenomena with other variables are frequently non-linear (e.g., inverse-U associations are not uncommon), and the corresponding true moderating effects may also be nonlinear and likely of higher order (e.g., three independent variables, instead of two, may simultaneously interact with one another to predict the outcome). In addition, moderators were often separately examined in different regression models (e.g., 70, 71, 139), each of which is an overly simplistic representation of the potentially complex interaction patterns. Such potential model misspecifications are expected to produce bias, invalid conclusions, and personalized treatment rules with suboptimal performance (140). It is important to note, however, that some of these issues are not specific to the reviewed research and are a result of 1) the small sample sizes of the behavioral trials and 2) the difficulty of implementing statistical methods that can address these issues (19).

Different statistical methods used across studies also contributed to the inconsistent results. For example, Wilson et al. (70) used a linear model with depression as a single predictor in interaction with treatment and did not find depression as a significant moderator of differential response. However, using the same sample, Sysko et al. (72) ran a latent class analysis on multiple measures including depression and found that the class including high levels of depression differentially predicted treatment response. Even in cases where significant moderating effects were identified, *post-hoc* probing was often not conducted or done for separate treatment conditions instead of different levels of the HiTOP dimension. As a result, it was not clear if the moderator was predictive (e.g., one of the treatments was always better but the effect may have been more pronounced at different levels of the moderator) or prescriptive (favoring one treatment at a certain level of the moderator and the other at another) (e.g., 141, 142).

A few of the reviewed studies addressed some of these issues (12, 59, 62, 73) with more advanced methods that can generate personalized treatment rules for optimal treatment selection. It should be noted that the flexibility of the methods in the reviewed articles varied widely. Several studies examined multiple moderator-treatment interactions and generated personalized treatment rules (62, 66, 141). However, because they were still based on standard linear regression approaches, they were likely prone to model misspecification and overfitting given small sample sizes. Some studies used machine learning approaches with no model assumptions for their variable selection procedure only, but these methods were almost always model-based in terms of the outcome under study and thus have similar parametric assumptions to linear regression (e.g., model-based recursive partitioning (73, 74, 100, 101, 143). Others combined multiple different variable selection procedures with varying flexibility on model assumptions (e.g., parametric linear methods and non-parametric that allow for non-linearity and complex interaction structures) (59, 73, 101). For example, some studies combined tree-based algorithms (e.g., random forest) with linear regression methods and selected only moderators that were consistently identified across both approaches. Others used spline smoothing that enables discovering non-linear patterns but did not allow for higher-order interactions (75). Most importantly, however, the personalized treatment rules that utilized these somewhat flexibly selected variables were, in most of these studies, generated based on standard linear regression (59, 73, 74, 101). Estimating individualized treatment rules based on such likely misspecified models, is expected to lead to recommendations that are inferior to the true optimal ones (140).

### 3.2 Recommendations for future research directions

Our goal was to provide preliminary evidence for the potential usefulness of HiTOP on differential treatment response to inform the viability of and catalyze future research using HiTOP dimensions for personalized treatment selection. Although the existing literature reviewed provides evidence for the potential usefulness of HiTOP dimensions of differing levels of specificity for personalized treatment selection that can guide future research, the field is still in its infancy and definite conclusions cannot be drawn. Also, the current state of the literature does not allow for aggregation of the findings and immediate use in clinical practice. The application of this line of work in clinical decision making will require extensive research before it can be properly translated and replicated. However, our review provided an extended picture of the state of the literature on the potential prescriptive role of HiTOP-relevant dimensions and revealed important routes for future research that can advance the field moving forward (for a summary of our recommendations, see **Table 3**). Although these conclusions are based on our scoping review, and thus are limited to internalizing disorders and CBTs, we believe these recommendations may generalize to other disorders and treatments as well and may serve as general research directions and priorities that will move the field of HiTOP-based applications to personalized treatment selection forward.

**Table 3:** Seven recommendations for advancing personalized mental health treatment selection with HiTOP.

1.	Use consistent, valid, and reliable measurement of HiTOP – e.g., HiTOP-friendly measures that have been proposed by the consortium until a validated HiTOP measure becomes available.
2.	Measure the entire or a large portion of the proposed hierarchical structure of psychopathology, including dimensions of multiple levels of specificity and spectrum belongingness.
3.	Use more diverse samples without strict exclusion criteria that shrink variability in psychopathology dimensions outside the spectrum of the primary diagnosis.
4.	Use smaller clinical trials as an intermediate step towards hypotheses-driven personalized treatment selection approaches as opposed to proposing guidelines for immediate use to clinical practice.
5.	Use statistical methods with minimal statistical assumptions based on modern frameworks of machine learning and causal inference that can address the issue of big data and can capture the complexity of interactions of individual characteristics on treatment response.
6.	Use larger randomized clinical trials as they provide the most rigorous evidence for personalized treatment rules because they protect against unmeasured confounding and supplement these trials with pragmatic trials and carefully designed, planned, and executed, large naturalistic prospective intervention studies as they can provide strong evidence about effectiveness in real life settings.
7.	Consider other individual characteristics (e.g., demographics, social determinants of health), domains of human functioning (that are relevant to psychopathology and can be relatively easily measured in clinical practice), and process variables that may affect treatment response in combination with psychopathology dimensions.

First, we recommend the use of consistent, valid, and reliable measurement of HiTOP dimensions in future research. Until a validated HiTOP measure becomes available, HiTOP-friendly measures have been proposed by the consortium that can be used in combination by researchers and clinicians (20, 144). This would increase considerably the reliability of HiTOP measurement across studies and enable more consistent, and valid measurement of homogenous HiTOP dimensions.

Second, we recommend measurement of the entire proposed hierarchical structure of psychopathology, including dimensions of multiple levels of specificity and spectrum belongingness. Only by this comprehensive examination we can draw conclusions concerning which specific parts of a psychopathology profile (higher vs. lower specificity dimensions and spectra) are important prescriptive factors for a given set of therapies and conditions treated. Interaction effects for such complex phenomena are expected to be of a quite complicated form. Any individual moderating effects may be misleading because, after accounting for the entire HiTOP structure, the direction of the identified interactions or even the significance might change. Also, some of the identified moderating effects of a given specificity level might reflect the true moderating effect of a lower specificity moderator that includes them (e.g., a shown significant moderating effect of insomnia might reflect the moderating effect of a general disposition to general psychopathology). Considering the entire structure will allow researchers to disentangle these relationships and potentially reveal personalized treatment rules with a better performance.

Third, to leverage the heterogeneity in this hierarchical psychopathology structure, we recommend using more diverse samples without strict exclusion criteria that shrink variability in psychopathology dimensions outside the spectrum of the sample’s primary diagnosis. This would result in higher variability in terms of putative moderator scores, which will more closely reflect clinical settings and can be leveraged to predict personalized response to treatment that will more likely be generalizable to real world settings.

Fourth, we caution against using results from traditional clinical trials examining individual moderating effects to propose immediate recommendations for clinical practice. The ability to capture the heterogeneity in psychopathology and complex moderating effects on treatment response is essential for precision mental health and the construction of personalized treatment rules for treatment selection. However, methodologically speaking, it comes at a cost. This heterogeneity equals to a large amount of data (19). To truly capture this heterogeneity, the ability to assess multiple moderators simultaneously is required, as well as the examination of complex and nonlinear relationships among the variables, and higher order treatment-moderator interactions (99, 145). Behavioral trials using traditional statistical approaches cannot handle this task because they rarely have enough power to detect significant moderating effects, and, even if they did, they cannot capture the complexity of these relationships. Therefore, conclusions from these trials should be considered preliminary regarding personalized treatment selection. They should not be used to propose guidelines for clinical practice but serve as an intermediate step towards hypotheses-driven personalized treatment selection approaches. Clearly, multiple comparisons and tests without correcting for Type I error often done in these trials would hinder this goal.

Fifth, flexible statistical methods with minimal statistical assumptions based on modern frameworks of machine learning and causal inference (19, 140, 145–147) can address these issues. For example, outcome weighted learning, residual weighted learning, or reinforcement learning are some flexible and rigorous methods for the estimation of personalized treatment rules (19, 148–150). These are one-stage methods (in contrast to those most often used for personalized mental health treatment selection), which offer a unified approach for (i) variable selection or/and regularization of the complexity of the rules, and (ii) derivation of model-free personalized treatment rules. They directly estimate a personalized treatment rule instead of imposing and estimating an often restrictive (and thus unrealistic) model for the entire outcome (a significant portion of which is not informative for precision medicine purposes).

Sixth, we recommend the use of large randomized clinical trials, as they would provide richer information, as well as the most rigorous evidence for personalized treatment rules, as they protect against unmeasured confounding (19). Trials with specialized designs developed to compare sequences of interventions individualized to patient characteristics (e.g., sequential multiple assignment randomized trial [SMART] designs) can be leveraged for adaptive treatment over time (27). Such designs can help identify which pathology could be addressed first and with which treatment, taking into account the unique picture of their client’s psychopathology and modifying recommendations over time based on response to the given treatment. However, it is important to note that given the strict exclusion criteria in clinical trials in general, samples are often highly specific and do not represent the entire typical patient population that clinicians will encounter. As such they give evidence about efficacy under ideal situations and to specific populations. Carefully designed, planned, and executed, large naturalistic prospective intervention studies or pragmatic trials may have the advantage of providing strong evidence about effectiveness in real life settings (19, 151).

Seventh, although it may be an important piece of information for personalized treatment selection, the psychopathology presentation is only part of the picture of a patient’s profile related to treatment outcomes (31). This may be one reason for the relatively small number of moderating effects identified in existing literature. An important route for future research is the consideration of other individual characteristics (e.g., sociodemographic factors), domains of human functioning (that are relevant to psychopathology and can be relatively easily measured in clinical practice), and process variables that may affect treatment response in combination with psychopathology dimensions. For example, “objective” behavioral task performance has shown to influence treatment outcomes. Similarly, psychotherapeutic process variables, such as therapeutic alliance, have been found to interact with other personal characteristics to influence response to treatment (152). Social determinants of health, such as economic stability, access to quality education and health care, and social/community context, are other individual characteristics that may influence treatment response. Such variables may interact with each other and psychopathology dimensions to influence treatment response and may be particularly important for adjusting ongoing treatment to the evolving needs of the client. If supported by research, this information can be incorporated in personalized treatment rules that can later be used by clinicians to enhance their treatment decisions based on their client’s profile and their evolving relationship. Finally, patient preferences as well as feasibility of receiving certain treatments due to health disparities and inequity in groups from diverse backgrounds are critical to consider. For example, about 1 in 10 people in the United States do not have health insurance (153), which limits access especially to long-term treatments. Preferences and accessibility can be taken into account in the constructed personalized treatment rule by relevant statistical methods (154).

## 4 Conclusion

In summary, theory and the existing literature reviewed provide evidence for the potential usefulness of HiTOP dimensions of differing levels of specificity for personalized treatment selection that can guide future research. We view the application of HiTOP-relevant dimensions to personalized mental health approaches as a viable and exciting direction in the field of mental health that offers many avenues for research for the improvement of patient outcomes. However, the field is still in its infancy and definite conclusions cannot be drawn, especially as heterogeneity of effects was high. Future work should address identified limitations in the field, including limited research in several HiTOP domains, strict inclusion/exclusion criteria shrinking individual heterogeneity, large variability in the measurement of HiTOP dimensions, potential risk of Type I and Type II error, and other methodological limitations for assessing personalized treatment response. Although the work to be done is vast, the payoff has the potential to be large. Research has begun using HiTOP-relevant dimensions to estimate personalized treatment rules that can eventually assist clinical decision-making for optimal treatment selection. These rules should next be tested using advanced statistical methods to optimize selection among sets of different treatment options, including different types of behavioral and pharmacological interventions, various modes of delivery, combinations of intervention, or different intervention timelines. If the promise of such rules is substantiated, and such rules are easy to apply clinically, they could optimize clinical decision making, maximizing benefits and minimizing costs for the individual client and the population as a whole.
