# Supplementary material for: Hierarchical taxonomy of psychopathology and personalized mental health treatment selection
Source: Front Psychiatry. 2025 Oct 29;16:1597879. doi: 10.3389/fpsyt.2025.1597879 (PMC12605141; doi:10.3389/fpsyt.2025.1597879)
Supplement: Supplementary file 1 [file SupplementaryFile1.docx]

**Appendix A**

**HiTOP-related dimensions terms**

- internalizing
- eating
- anxiety, anxious
- compulsion, compulsive
- depression, depressive)
- dysphoria
- obsessive
- phobia, phobic
- agoraphobia
- PTSD, posttraumatic stress disorder)
- panic
- posttraumatic

**Moderator analysis, precision medicine/mental health-related terms**

- moderation, moderating, moderator
- differential predictor
- interaction, interacting, interactive
- precision medicine
- personalized medicine
- precision mental health
- personalized mental health
- optimal treatment rules
- individualized treatment rules
- optimal treatment regimes
- dynamic treatment regimes
- dynamic treatment rules
- clinical placement guidelines
- personalized treatment selection
- precision treatment selection
- individualized treatment selection
- individualized treatment
- precision treatment rules
- effect modifier
- effect modification

**Psychotherapy Interventions**

- cognitive behavioral therapy, CBT
- cognitive therapy
- acceptance and commitment therapy, ACT
- dialectical behavior therapy, DBT
- behavioral activation
- mindfulness, mindfulness-based interventions

**Exclusions: Medical / Neurological Conditions**

(to exclude in **Title** or **Keywords**)

- cancer
- cardiovascular
- heart disease
- diabetes
- intracranial aneurysm
- traumatic brain injury
- HIV
- autism
- fibromyalgia
- Parkinson’s disease
- Alzheimer’s disease
- tinnitus
- multiple sclerosis
- hemophilia
- chronic pain

**Exclusions: Publication Types**

(to exclude in **Title**)

- meta-analysis
- review
- protocol
- meta-analytic
- case study
- case report

**Appendix B**

**Table B.1**

*Studies included in the systematic review, study characteristics, and findings*

B**.1.1 Treatment for distress disorders**

| **Study** | **Study Characteristics** | **Treatments** | **Outcome** | **Moderator** | **Spectrum** | **Moderator Characteristics** | **Result** |
| --- | --- | --- | --- | --- | --- | --- | --- |
| Barber et al., 1996 | Outpatients with current MDD episode and >13 on HDRS, N=235, Country=United States, Design=RCT | 1) CT, 2) IPT | Depression severity (HRSD, BDI) | Avoidant PD (PAF) | Detachment | Specificity=3, Fit=1 | **Higher level:** CT outperformed IPT post-treatment  **Lower level:** IPT outperformed CT post-treatment |
|  |  |  |  |  |  |  |  |
|  |  |  |  | Depression (BDI) | Internalizing | Specificity=3, Fit=1 | **Higher level:** IPT outperformed CT post-treatment **Lower level:** no difference |
|  |  |  |  |  |  |  |  |
|  |  |  |  | Obsessive PD (PAF) | Internalizing | Specificity=3, Fit=0 | **Higher level:** IPT outperformed CT post-treatment **Lower level:** CT outperformed IPT post-treatment |
|  |  |  |  |  |  |  |  |
| Bernecker et al., 2016 | Adults with MDD, N=69, Mage=38.67, F=75.36, Non-White=17.05, Country=Canada, Design=RCT | 1) CBT, 2) IPT | Depression severity (BDI-II, HamD-6) | Intimacy Avoidance (RSQ) | Detachment | Specificity=4, Fit=1 | (--) |
|  |  |  |  | Intimacy Avoidance (ECR-R) | Detachment | Specificity=4, Fit=1 | (--) |
|  |  |  |  | Separation insecurity (ECR-R) | Internalizing | Specificity=4, Fit=0 | (--) |
|  |  |  |  | Separation insecurity (RSQ) | Internalizing | Specificity=4, Fit=0 | (--) |
| Blatt et al., 1995 | Outpatients with current MDD episode, N=239, Mage=35, F=70, Non-White=11, Country=United States, Design=RCT | 1) CBT, 2) IPT, 3) Clinical management with placebo | Depression severity (BDI, , HRSD), General psychopathology (SCL-90) | Perfectionism (low) (DAS) | Disinhibited Externalizing | Specificity=4, Fit=1 | (--) |
|  |  |  |  |  |  |  |  |
|  |  |  |  |  |  |  |  |
| Bulmash et al., 2009 | Adults with MDD, N=113, Mage=43, F=65.49, Country=Canada, Design=RCT | 1) CBT, 2) IPT, 3) Medication | 50% Reduction in depressive symptoms (HRSD) | Separation Insecurity (Dependency - DEQ) | Internalizing | Specificity=4, Fit=0 | (--) |
|  |  |  |  |  |  |  |  |
|  |  |  |  | Ineptitude (Self-criticism - DEQ) | Internalizing | Specificity=4, Fit=1 | (--) |
| Carter et al., 2011 | Adults with MDD, N=177, Mage=35.2, F=72.3, Country=New Zealand, Design=RCT | 1) CBT, 2) IPT | Depression severity (MADRS) | General psychopathology (DSM-IV general personality pathology symptoms) | General Psychopathology | Specificity=0, Fit=1 | **Higher level:** CBT outperformed IPT in percent improvement post-treatment **Lower level:** No significant difference |
|  |  |  |  | Depression (MADRS) | Internalizing | Specificity=3, Fit=1 | (--) |
| Carter et al., 2018 | Outpatients with MDD, N=100, Mage=38, F=69, Country=New Zealand, Design=RCT | 1) CBT, 2) Schema Therapy | Depression severity (MADRS) | General psychopathology (SCID-II) | General Psychopathology | Specificity=0, Fit=1 | (--) |
|  |  |  |  |  |  |  |  |
|  |  |  |  | Depression (MADRS) | Internalizing | Specificity=3, Fit=1 | (--) |
| Cohen et al., 2019 | Outpatients with MDD, N=167, Mage=39.6, F=71, Country=Netherlands, Design=RCT | 1) CBT, 2) Psychodynamic Psychotherapy | Depression severity (HRSD) | Aggression (LEIDS) | Antagonstic Externalizing | Specificity=4, Fit=0 | (--) |
|  |  |  |  | Extraversion (NEO) | Detachment | Specificity=1, Fit=1 | **Higher level:** CBT outperformed psychodynamic psychotherapy, post-treatment **Lower level:** Psychodynamic psychotherapy outperformed CBT, post-treatment |
|  |  |  |  |  |  |  |  |
|  |  |  |  | Extraversion (NVM) | Detachment | Specificity=1, Fit=1 | (--) |
|  |  |  |  | Perfectionism (low) (LEIDS) | Disinhibited Externalizing | Specificity=4, Fit=0 | (--) |
|  |  |  |  | Risk taking (Risk aversion - LEIDS) | Disinhibited Externalizing | Specificity=4, Fit=0 | (--) |
|  |  |  |  | Neuroticism (NEO) | Internalizing | Specificity=1, Fit=1 | (--) |
|  |  |  |  | Depression (BSI) | Internalizing | Specificity=3, Fit=1 | **Higher level:** CBT outperformed psychodynamic psychotherapy post-treatment **Lower level:** Psychodynamic psychotherapy outperformed CBT post-treatment |
|  |  |  |  |  |  |  |  |
|  |  |  |  | Depression (HDRS) | Internalizing | Specificity=3, Fit=1 | (--) |
|  |  |  |  | Anxiety (BAI) | Internalizing | Specificity=3, Fit=1 | (--) |
|  |  |  |  | Anxiety sensitivity (ASI) | Internalizing | Specificity=4, Fit=1 | **Higher level:** Psychodynamic psychotherapy outperformed CBT, post-treatment **Lower level:** CBT outperformed psychodynamic psychotherapy, post-treatment |
|  |  |  |  |  |  |  |  |
|  |  |  |  | Behavior restricting fears (BSI) | Internalizing | Specificity=4, Fit=1 | (--) |
|  |  |  |  | Hopelessness (LEIDS) | Internalizing | Specificity=4, Fit=1 | (--) |
|  |  |  |  | Hostility (BSI) | Internalizing | Specificity=4, Fit=1 | (--) |
|  |  |  |  | Somatization (NVM) | Somatoform | Specificity=4, | (--) |
|  |  |  |  |  |  | Fit=0 |  |
|  |  |  |  | Cognitive complaints (BSI) | Somatoform | Specificity=4, Fit=0 | (--) |
|  |  |  |  | Somatization (BSI) | Somatoform | Specificity=4, Fit=0 | (--) |
|  |  |  |  | Thought disorder (BSI) | Thought Disorder | Specificity=1, Fit=1 | (--) |
|  |  |  |  | Paranoid ideation (BSI) | Thought Disorder | Specificity=4, Fit=1 | (--) |
| Coyne et al., 2022 | Adults with MDD, N=80, Mage=38.75, F=71.24, Country=Canada, Design=RCT | 1) CBT, 2) IPT | Depression severity (BDI-II) | General personality pathology (SCID-II) | General Psychopathology | Specificity=0, Fit=1 | (--) |
|  |  |  |  | Thought disorder (SCID-II) | Thought Disorder | Specificity=1, Fit=1 | (--) |
|  |  |  |  | Avoidant PD (SCID-II) | Detachment | Specificity=3, Fit=1 | (--) |
|  |  |  |  | Obsessive PD (SCID-II) | Internalizing | Specificity=3, Fit=0 | (--) |
|  |  |  |  | Self-sacrificing (IIP) | Internalizing | Specificity=4, Fit=0 | **Higher level:** CBT outperformed IPT   **Lower level:** IPT outperformed CBT |
| de Graaf et al., 2010 | Adults with depressive symptoms, N=303, Mage=44.87, F=56.31, Country=Netherlands, Design=RCT | 1) Computerized unguided CBT, 2) TAU, 3) CBT + TAU | Depression severity (BDI-II) | General psychopathology (SCL-90) | General Psychopathology | Specificity=0, Fit=1 | (--) |
|  |  |  |  | Depression (BDI-II) | Internalizing | Specificity=3, Fit=1 | (--) |
|  |  |  |  | Separation Insecurity (Dependency - DAS-A) | Internalizing | Specificity=4, Fit=0 | (--) |
|  |  |  |  | Ineptitude (DAS-A) | Internalizing | Specificity=4, Fit=1 | (--) |
| Deisenhofer et al., 2018 | Patients with PTSD, N=317, Mage=40.21, F=57.78 Country=United Kingdom, Design=Retrospective | 1) Trauma-focused CBT, 2) EMDR | Depression severity (PHQ-9) | Depression (PHQ-9) | Internalizing | Specificity=3, Fit=1 | **EMDR**: for higher level of depression the effect was larger than for lower level  **CBT**: no difference across levels |
|  |  |  |  |  |  |  |  |
|  |  |  |  | Generalized anxiety (GAD-7) | Internalizing | Specificity=4, Fit=1 | (--) |
| Delgadillo et al., 2020 | Patients treated for depression in a primary care service, N=1,435, Mage=39.64, F=64.4, Non-White=11.8, Country=United Kingdom, Design=Retrospective | 1) CBT, 2) Person-centered Counseling | Depression severity (PHQ-9) | Depression (PHQ-9) | Internalizing | Specificity=3, Fit=1 | (--) |
|  |  |  |  |  |  |  |  |
|  |  |  |  | Generalized anxiety (GAD-7) | Internalizing | Specificity=4, Fit=1 | (--) |
| Driessen et al., 2016 | Outpatients with MDD episode, N=233, Mage=39.98, F=67.8, Non-White=8.8, Country=Netherlands, Design=RCT | 1) CBT, 2) Short-term psychodynamic supportive psychotherapy (SPSP) | Depressive severity (HDRS) | General psychopathology (BSI) | General Psychopathology | Specificity=0, Fit=1 | (--) |
|  |  |  |  | Depression (HDRS) | Internalizing | Specificity=3, Fit=1 | **Moderate depression + psychotherapy only + low anxiety:** SPSP outperformed CBT |
|  |  |  |  |  |  |  | **Severe depression + psychotherapy + medication who reported a duration of the depressive episode of ≥1 year:** SPSP outperformed CBT |
|  |  |  |  |  |  |  | **Severe depression + psychotherapy + medication who reported a duration of the depressive episode of <1 year**: CBT outperformed SPSP |
|  |  |  |  | Anxiety (BAI) | Internalizing | Specificity=3, Fit=1 | **Moderate depression + psychotherapy only + low anxiety:** SPSP outperformed CBT |
|  |  |  |  | Anxiety sensitivity (ASI) | Internalizing | Specificity=4, Fit=1 | (--) |
| Ehlers et al., 2023 | Aduls who met criteria for ICD-11 complex PTSD N=217, Country=UK, Mage=36.36, F=73, Non-White=13, Design=RCT | 1) Internet guided Trauma-focused CBT, 2) Internet guided CBT | PTSD symptoms (PCL-5) | Dissociation (TSDQ) | Internalizing | Specificity=4, Fit=1 | Higher level associated with greater advantage of Internet guided Trauma-focused CBT over Internet guided CBT than lower level |
| Friedl et al., 2019 | Depressive outpatients, N=123, Country=Switzerland, Design=RCT | 1) CBT, 2) CBT with integrated exposure and emotion-focused elements | Depression severity (BDI-II) | General psychopathology (SCL-K-9) | General Psychopathology | Specificity=0, Fit=1 | **Integrated CBT:** Lower level better response than higher level post-treatment |
|  |  |  |  |  |  |  | **Standard CBT:** no difference across levels |
|  |  |  |  | Avoidance (CBAS) | Internalizing | Specificity=4, Fit=1 | (--) |
|  |  |  |  | Ineptitude (Self-efficacy; RSE) | Internalizing | Specificity=4, Fit=1 | (--) |
|  |  |  |  | Ineptitude (Self-esteem; SWE) | Internalizing | Specificity=4, Fit=1 | (--) |
|  |  |  |  | Ineptitude (DAS) | Internalizing | Specificity=4, Fit=1 | (--) |
|  |  |  |  | Dominance (IIP-32) | Antagonstic Externalizing | Specificity=4, Fit=0 | (--) |
| Gomez Penedo et al., 2017 | Patients with a principal GAD diagnosis, N=85, Mage=33.33, F=88.24, Non-White=24.71, Country=Canada, Design=RCT | 1) CBT, 2) CBT with motivational interviewing (MI-CBT) | Worry (PSWQ) | Dominance (IIP-C) | Antagonstic Externalizing | Specificity=4, Fit=0 | **Lower level:** MI-CBT outperformed CBT in worry reduction **Higher level:** no difference between treatments in worry reduction |
|  |  |  |  |  |  |  |  |
|  |  |  |  | Submissiveness (Exploitable - IIP-C) | Internalizing | Specificity=4, Fit=0 | (--) |
|  |  |  |  | Submissiveness (Nonassertive - IIP-C) | Internalizing | Specificity=4, Fit=0 | **Higher level:** greater worry reduction in MI-CBT versus CBT |
|  |  |  |  |  |  |  | **Lower level:** no difference between treatments in worry reduction |
| Huibers et al., 2015 | Adult outpatients with a primary diagnosis of MDD, N=134, Mage=41.25, F=66.23, Country=Netherlands, Design=RCT | 1) CT, 2) IPT | Depression severity (BDI-II) | Aggression (LEIDS) | Antagonistic Externalizing | Specificity=4, Fit=0 | (--) |
|  |  |  |  | Dominance (IIP) | Antagonistic Externalizing | Specificity=4, Fit=0 | (--) |
|  |  |  |  | Attention seeking (Intrusive/Needy - IIP) | Antagonistic Externalizing | Specificity=4, Fit=0 | (--) |
|  |  |  |  | Vindictiveness (IIP) | Antagonistic Externalizing | Specificity=4, Fit=1 | (--) |
|  |  |  |  | Social withdrawal (Socially avoidant - IIP) | Detachment | Specificity=4, Fit=0 | (--) |
|  |  |  |  | Disaffiliativeness (Cold/distant - IIP) | Detachment | Specificity=4, Fit=0 | (--) |
|  |  |  |  | Perfectionism (low) (LEIDS) | Disinhibited Externalizing | Specificity=4, Fit=0 | (--) |
|  |  |  |  | Risk taking (Risk aversion - LEIDS) | Disinhibited Externalizing | Specificity=4, Fit=0 | (--) |
|  |  |  |  | Depression (BSI) | Internalizing | Specificity=3, Fit=1 | (--) |
|  |  |  |  | Anxiety (BSI) | Internalizing | Specificity=3, Fit=1 | (--) |
|  |  |  |  | Submissiveness (Exploitable - IIP) | Internalizing | Specificity=4, Fit=0 | (--) |
|  |  |  |  | Submissiveness (Nonassertive - IIP) | Internalizing | Specificity=4, Fit=0 | (--) |
|  |  |  |  | Submissiveness (Self-sacrificing - IIP) | Internalizing | Specificity=4, Fit=0 | **Higher level:** CT outperformed IPT, at post-treatment |
|  |  |  |  | Behavior restricting fears (BSI) | Internalizing | Specificity=4, Fit=1 | (--) |
|  |  |  |  | Hopelessness (LEIDS) | Internalizing | Specificity=4, Fit=1 | (--) |
|  |  |  |  | Hostility (BSI) | Internalizing | Specificity=4, Fit=1 | (--) |
|  |  |  |  | Ineptitude (SLSC-R) | Internalizing | Specificity=4, Fit=1 | (--) |
|  |  |  |  | Ineptitude (BSI) | Internalizing | Specificity=4, Fit=1 | (--) |
|  |  |  |  | Ineptitude (SLSC-R) | Internalizing | Specificity=4, Fit=1 | (--) |
|  |  |  |  | Cognitive complaints (BSI) | Somatoform | Specificity=4, Fit=0 | **Higher level:** IPT outperformed CT, at post-treatment |
|  |  |  |  | Somatization (BSI) | Somatoform | Specificity=4, Fit=0 | **Higher level:** CT outperformed IPT, at post-treatment |
|  |  |  |  | Thought disorder (BSI) | Thought Disorder | Specificity=1, Fit=1 | (--) |
|  |  |  |  | Paranoid ideation (BSI) | Thought Disorder | Specificity=4, Fit=1 | **Higher level:** CT outperformed IPT, at post-treatment |
| Kay-Lambkin et al., 2017 | Adults with concurrent depression and alcohol/cannabis misuse, N=274, Mage=40, F=43, Country=Australia, Design=RCT | 1) CBT + MI, 2) Internet guided CBT + MI, 3) Person-centered Counseling | Alcohol use, Cannabis use (OTI), Depression severity (BDI-II) | Perfectionism (DAS) | Disinhibited Externalizing | Specificity=4, Fit=1 | Higher level: better response to computer-delivered CBT+MI than therapist CBT from 3 to 12-month follow-up on depression outcome |
|  |  |  |  |  |  |  |  |
|  |  |  |  | Need for approval (DAS) | Internalizing | Specificity=4, Fit=1 | (--) |
| Lemmens et al., 2015 | Adult outpatients with DSM-IV MDD, N=182, Mage=41.2, F=66.2, Country=Netherlands, Design=RCT | 1) CT, 2) IPT, 3) Waitlist | Depression severity (BDI-II), General psychopathology (BSI), Quality of life (Work and Social Adjustment Scale, RAND-36) | Depression (BDI-II) | Internalizing | Specificity=3, Fit=1 | (--) |
|  |  |  |  |  |  |  |  |
|  |  |  |  |  |  |  |  |
| Littleton et al., 2016 | College women with rape-related PTSD, N=87, Mage=22, F=100, Non-White=54.3, Country=United States, Design=RCT | 1) Internet guided CBT, 2) Internet unguided Psychoeducation | Depression severity (CES-D), Anxiety symptoms (FDAS), PTSD symptoms (PSS-I) | Fear (FDAS) | Internalizing | Specificity=2, Fit=1 | (--) |
|  |  |  |  | Depression (CES-D) | Internalizing | Specificity=3, Fit=1 | (--) |
|  |  |  |  | PTSD (PSS-I) | Internalizing | Specificity=3, Fit=1 | **Lower level:** Psychoeducation outperformed CBT in PTSD symptom reduction at 3-month follow-up |
|  |  |  |  |  |  |  | **Higher level:** non-significant opposite trend in PTSD symptoms at 3-month follow-up |
| Lopez-Gomez et al., 2019 | Women with a DSM-IV diagnosis of MDD or dysthymia, N=128, Mage=52.02, F=100, Country=Spain, Design=non-RCT | 1) Group CBT, 2) Group Integrative Positive Psychology Intervention for Depression (IPPI-D) | Depression severity (BDI-II) | Negative affectivity (PANAS) | Internalizing | Specificity=1, Fit=1 | (--) |
|  |  |  |  | Anxiety (BAI) | Internalizing | Specificity=3, Fit=1 | (--) |
|  |  |  |  | Positive affect (low) (PANAS) | Internalizing | Specificity=4, Fit=0 | (--) |
|  |  |  |  | Positive relations (low) (PWBS) | Internalizing | Specificity=4, Fit=1 | (--) |
|  |  |  |  | Activation (BAS) | Internalizing | Specificity=4, Fit=0 | (--) |
| Lorenzo-Luaces et al., 2017 | Patients with MDD, N=622, Mage=36.36, F=62.32, Country=Netherlands, Design=RCT | 1) High intensity CBT, 2) Brief Therapy, 3) TAU | Recovery (absence of MDD status) | Agreeableness (NEO) | Antagonistic Externalizing | Specificity=1, Fit=1 | (--) |
|  |  |  |  | Extraversion (NEO) | Detachment | Specificity=1, Fit=1 | Integrated into prognostic index that predicted better response to CBT than brief therapy or TAU for those with poorer prognosis (including higher introversion) at the 18–24-month follow-up |
|  |  |  |  | Neuroticism (NEO) | Internalizing | Specificity=1, Fit=1 | (--) |
|  |  |  |  | Depression (SCL-90) | Internalizing | Specificity=3, Fit=1 | Integrated into prognostic index that predicted better response to CBT than brief therapy or TAU for those with poorer prognosis (including higher introversion) at the 18–24-month follow-up |
|  |  |  |  | Hostility (SCL-90) | Internalizing | Specificity=4, Fit=1 | Integrated into prognostic index that predicted better response to CBT than brief therapy or TAU for those with poorer prognosis (including higher introversion) at the 18–24-month follow-up |
|  |  |  |  | Openess (NEO) | Thought Disorder | Specificity=1, Fit=1 | (--) |
|  |  |  |  | Insomnia (Sleep complaints - SCL-90) | Internalizing | Specificity=4, Fit=1 | Integrated into prognostic index that predicted better response to CBT than brief therapy or TAU for those with poorer prognosis (including higher introversion) at the 18–24-month follow-up |
| McBride et al., 2006 | Patients with MDD, N=56, Mage=40.1 (IPT) / 41 (CBT), F=74% (IPT), 72.4% (CBT), Country=Canada, Design=RCT | 1) CBT, 2) IPT | Depression severity (BDI-II, Ham-D9), MDD remission (HDRS) | Avoidant PD (SCID–II/PQ) | Detachment | Specificity=3, Fit=1 | (--) |
|  |  |  |  | Intimacy Avoidance (RSQ) | Detachment | Specificity=4, Fit=1 | **Higher level:**  CBT outperformed IPT post-treatment on all outcomes |
|  |  |  |  | Obsessive PD (SCID–II/PQ) | Internalizing | Specificity=3, Fit=0 | (--) |
|  |  |  |  | Separation insecurity (RSQ) | Internalizing | Specificity=4, Fit=0 | (--) |
| McCarter et al., 2016 | Adults with co-occurring alcohol misuse and depression, N=290, Mage=44.58, F=45, Country=Australia, Design=RCT | 90-minute brief intervention and one of the following groups: 1) No treatment 2) Integrated (alcohol- and depression-focused) therapy (delivered either by a therapist or computer program), 3) Alcohol-focused therapy, 4) Depression-focused therapy | Change in alcohol use (OTI), Depression severity (BDI-II), Global Functioning (GAF) | General Psychopathology (General personality pathology -IPDE) | General Psychopathology | Specificity=0, Fit=1 | **Higher level:** integrated interventions were relatively more effective than single-focused ones |
|  |  |  |  | Detachment (IPDE) | Detachment | Specificity=1, Fit=1 | (--) |
|  |  |  |  | Antagonistic EXT (IPDE) | Antagonistic Externalizing | Specificity=1, Fit=1 | (--) |
|  |  |  |  | Thought disorder (IPDE) | Thought Disorder | Specificity=1, Fit=1 | **Higher level:** integrated interventions were relatively more effective than single-focused ones (marginally significant due to multiple comparisons) |
| Newman et al., 2017 | Adults with a principal diagnosis of GAD, N=47, Mage=39.11, F=63.8, Non-White=10.6, Country=United States, Design=RCT | 1) CBT, 2) CT, 3) Behavior Therapy | Generalized anxiety symptom severity (CSR, STAI, HARS, PSWQ) | Domineering (IIP-C) | Antagonistic Externalizing | Specificity=4, Fit=0 | **Higher level:** Behavior therapy outperformed CBT and CT at every follow-up |
|  |  |  |  | Attention seeking (Intrusive/Needy - IIP-C) | Antagonistic Externalizing | Specificity=4, Fit=0 | **Higher level:** Behavior therapy outperformed CBT and marginally CT |
|  |  |  |  |  |  |  | No difference between CBT and CT |
|  |  |  |  | Vindictiveness (IIP-C) | Antagonistic Externalizing | Specificity=4, Fit=1 | (--) |
|  |  |  |  | Disaffiliativeness (Cold/distant - IIP-C) | Detachment | Specificity=4, Fit=0 | (--) |
|  |  |  |  | Social withdrawal (Socially avoidant - IIP-C) | Detachment | Specificity=4, Fit=0 | (--) |
|  |  |  |  | Submissiveness (Exploitable - IIP-C) | Internalizing | Specificity=4, Fit=0 | (--) |
|  |  |  |  | Submissiveness (Nonassertive - IIP-C) | Internalizing | Specificity=4, Fit=0 | (--) |
|  |  |  |  | Submissiveness (Self-sacrificing - IIP-C) | Internalizing | Specificity=4, Fit=0 | (--) |
| Newman et al., 2019 | Individuals meeting criteria for DSM-III-R GAD N=66, Mage=37, F=65.15, Non-White=9.09, Design=RCT | 1) CBT, 2) Nondirective psychotherapy, 3) Applied Relaxation | Daily anxiety rating (0– 100 scale), anxiety severity (HARS, CSR) | Depression (HDRS) | Internalizing | Specificity=3, Fit=1 | **Higher level:** CBT outperformed nondirective therapy No difference was observed between CBT and appled relaxation |
| Nicholas et al., 2021 | Adults with Depression N=304, Mage=37.6, F=73.4, Non-White=11.5, Country=United States, Design=RCT | 1) Telephone-based CBT, 2) Internet guided CBT | Depression severity (PHQ-9) | Generalized anxiety (GAD-7) | Internalizing | Specificity=4, Fit=1 | (--) |
| Pedrelli et al., 2020 | College students with heavy episodic drinking and depressive symptoms, N=94, Mage=19.9, F=68.1, Non-White=45.7, Country=United States, Design=RCT | 1) CBT, 2) CBT + MI | Alcohol related problems (past month) (B-YAACQ), Depressive symptoms (BDI), Heavy episodic drinking (past month) (TLFB) | Alcohol use (TLFB) | Disinhibited Externalizing | Specificity=4, Fit=1 | **Lower level:** CBT for depression marginally outperformed MI+CBT in alcohol problems reduction |
|  |  |  |  |  |  |  |  |
|  |  |  |  | Depression (BDI) | Internalizing | Specificity=3, Fit=1 | **Lower level:** CBT for depression outperformed CBT+MI in heavy alcohol use reduction at 1-month follow-up only, not post-treatment  **Higher level**: no difference between conditions |
| Pots et al.,2016 | Adults from the general population with mild to moderate depressive symptoms, N=236, Mage=46.85, F=75.8, Country=Netherlands, Design=RCT | 1) Internet guided ACT, 2) Internet guided Expressive Writing Intervention, 3) Waitlist | Depression severity (CES-D) | Anxiety (HADS-A) | Internalizing | Specificity=3, Fit=1 | (--) |
|  |  |  |  | Depression (CES-D) | Internalizing | Specificity=3, Fit=1 | (--) |
|  |  |  |  | Well-being (MHC-SF) | Internalizing | Specificity=4, Fit=1 | (--) |
| Probst et al., 2020 | Patients with a DSM-IV current major depressive episode N=68, Mage=49.56, F=61.8, Country=Germany, Design=RCT | 1) Group MBCT, 2) Group CBASP | Depressive symptoms (HAM-D, BDI-II) | Domineering (IIP-32) | Antagonistic Externalizing | Specificity=4, Fit=0 | (--) |
|  |  |  |  | Attention seeking (Intrusive/Needy - IIP-32) | Antagonistic Externalizing | Specificity=4, Fit=0 | (++++) |
|  |  |  |  | Vindictiveness (IIP-32) | Antagonistic Externalizing | Specificity=4, Fit=1 | **Higher level**: MBCT outperformed CBASP for both outcomes at 6-month follow-up **Lower level:** no difference |
|  |  |  |  |  |  |  |  |
|  |  |  |  | Disaffiliativeness (Cold/distant - IIP-32) | Detachment | Specificity=4, Fit=0 | (--) |
|  |  |  |  | Social withdrawal (Socially avoidant - IIP-32) | Detachment | Specificity=4, Fit=0 | (--) |
|  |  |  |  | Submissiveness (Exploitable - IIP-32) | Internalizing | Specificity=4, Fit=0 | (--) |
|  |  |  |  | Submissiveness (Nonassertive - IIP-32) | Internalizing | Specificity=4, Fit=0 | **Higher level**: CBASP outperformed MBCT for both outcomes at 6-month follow-up **Lower level:** no difference |
|  |  |  |  |  |  |  |  |
|  |  |  |  | Submissiveness (Self-sacrificing - IIP-32) | Internalizing | Specificity=4, Fit=0 | (--) |
| Quilty et al., 2008 | Outpatients with a DSM-IV MDD diagnosis, N=649, Mage=39.81, F=66.9, Country=France, Design=Non-RCT | 1) CBT, 2) Psychodynamic Psychotherapy, 3) Supportive Therapy | >=50% decrease in depressive symptoms (MADRS) | Agreeableness (D5D) | Antagonistic Externalizing | Specificity=1, Fit=1 | (--) |
|  |  |  |  | Extraversion (D5D) | Detachment | Specificity=1, Fit=1 | (--) |
|  |  |  |  | Consiensciousness (D5D) | Disinhibited Externalizing | Specificity=1, Fit=1 | (--) |
|  |  |  |  | Neuroticism (D5D) | Internalizing | Specificity=1, Fit=1 | (--) |
|  |  |  |  | Openness (D5D) | Thought Disorder | Specificity=1, Fit=1 | (--) |
| Resick et al., 2012 | Adult women with PTSD, secondary to an index event of a sexual or physical assault in childhood or adulthood, N=150, Mage=35.44, F=100, Non-White=37.9, Country=United States, Design=RCT | 1) CT, 2) CPT, 3) Written Trauma Account | PTSD symptoms (PDS) | Depersonalization (MDI) | Internalizing | Specificity=4, Fit=1 | **Higher level:** CPT outperformed CT over treatment and 6- and 12-month follow-up |
|  |  |  |  | Derealization (MDI) | Internalizing | Specificity=4, Fit=1 | (++++) |
|  |  |  |  | Dissociation (TSI) | Internalizing | Specificity=4, Fit=1 | Non-linear significant interaction over treatment and 6- and 12-month follow-up  **Lower leve**l: CT outperformed CPT **Higher level**: CPT outperformed CT |
|  |  |  |  | Dissociative disengagement (MDI) | Internalizing | Specificity=4, Fit=1 | Non-linear significant interaction over treatment and 6- and 12-month follow-up  **Lower level:** CT outperformed CPT **Higher level:** CPT outperformed CT |
|  |  |  |  | Emotional constriction (MDI) | Internalizing | Specificity=4, Fit=1 | (--) |
|  |  |  |  | Memory disturbances (MDI) | Internalizing | Specificity=4, Fit=1 | (--) |
|  |  |  |  | Multiplicity (MDI) | Internalizing | Specificity=4, Fit=1 | (--) |
|  |  |  |  | Trauma-specific dissociation (MDI) | Internalizing | Specificity=4, Fit=1 | Non-linear significant interaction over treatment and 6- and 12-month follow-up |
|  |  |  |  |  |  |  | **Lower level:** CT outperformed CPT |
|  |  |  |  |  |  |  | **Higher level:** CPT outperformed CT |
| Resick et al., 2020 | Active duty service members with PTSD, N=165, Mage=34.2, F=9, Non-White=58, Country=United States, Design=RCT | 1) Individual CPT, 2) Group CPT | PTSD symptom severity (PSS-I) | Anxiety (BAI) | Internalizing | Specificity=3, Fit=1 | (--) |
|  |  |  |  | Depression (BDI-II) | Internalizing | Specificity=3, Fit=1 | (--) |
|  |  |  |  | Insomnia (ISI) | Internalizing | Specificity=4, Fit=1 | (--) |
|  |  |  |  | Suicidality (BSSI) | Internalizing | Specificity=4, Fit=1 | (--) |
| van Bronswijk et al., 2018 | Adult outpatients with DSM-IV MDD, N=146, Country=Netherlands, Design=RCT | 1) CT, 2) IPT | Depressive symptoms (BDI-II) | Antagonistic EXT (SCID-II) | Antagonistic Externalizing | Specificity=1, Fit=1 | (--) |
|  |  |  |  | Avoidant PD (SCID-II) | Detachment | Specificity=3, Fit=1 | (--) |
|  |  |  |  | Dependent PD (SCID-II) | Detachment | Specificity=3, Fit=0 | (--) |
|  |  |  |  | Obsessive PD (SCID-II) | Internalizing | Specificity=3, Fit=0 | (--) |
|  |  |  |  | Thought disorder (SCID-II) | Thought Disorder | Specificity=1, Fit=1 | Significant interaction |
|  |  |  |  |  |  |  | **IPT:** higher levels less decrease in depression than lower levels |
|  |  |  |  |  |  |  | **CT:** no difference across levels |
| Van Bronswijk et al., 2019 | Adult outpatients with DSM-IV MDD, N=151, F=66.2, Country=Netherlands, Design=RCT | 1) CT, 2) IPT | Depressive symptoms (BDI-II) | Anxiety (BSI) | Internalizing | Specificity=3, Fit=1 | (--) |
|  |  |  |  | Behavior restricting fears (BSI) | Internalizing | Specificity=4, Fit=1 | (--) |
|  |  |  |  | Depression (BSI) | Internalizing | Specificity=3, Fit=1 | (--) |
|  |  |  |  | Hopelessness (BHS) | Internalizing | Specificity=4, Fit=1 | (--) |
|  |  |  |  | Hostility (BSI) | Internalizing | Specificity=4, Fit=1 | (--) |
|  |  |  |  | Ineptitude (SLSC-R) | Internalizing | Specificity=4, Fit=1 | (--) |
|  |  |  |  | General interpersonal distress (IIP) | General Psychopathology | Specificity=0, Fit=0 | (--) |
|  |  |  |  | Cognitive complaints (BSI) | Somatoform | Specificity=4, Fit=0 | (--) |
|  |  |  |  | Somatization (BSI) | Somatoform | Specificity=4, Fit=0 | (--) |
|  |  |  |  | Paranoid ideation (BSI) | Thought Disorder | Specificity=4, Fit=1 | (--) |
| Warmerdam et al., 2013 | Adults with depressive symptoms, N=263, Mage=45.1, F=71.1, Country=Netherlands, Design=RCT | 1) Internet guided CBT, 2) Internet guided Problem-solving therapy, 3) Waitlist | Depression severity (CES-D) | Impulsivity (SPSI-R) | Disinhibited Externalizing | Specificity=4, Fit=0 | (++++) |
|  |  |  |  | Anxiety (HADS-A) | Internalizing | Specificity=3, Fit=1 | (++++) |
|  |  |  |  | Avoidance (SPSI-R) | Internalizing | Specificity=4, Fit=1 | (++++) |
|  |  |  |  | Depression (CES-D) | Internalizing | Specificity=3, Fit=1 | (--) |

**B.1.2. Treatment for eating disorders**

| **Study** | **Study Characteristics** | **Treatments** | **Outcome** | **Moderator** | **Spectrum** | **Moderator Characteristics** | **Result** |
| --- | --- | --- | --- | --- | --- | --- | --- |
| Accurso et al., 2016 | Adults with DSM-IV bulimia nervosa or bulimia nervosa symptoms not meeting diagnostic criteria, N=80, Mage=27.3, F=90, Non-White=12.5, Country=United States, Design=RCT | 1) CBT-E, 2) Integrative Cognitive-Affective Therapy (ICAT) | EDE | Intimacy Avoidance (DAPP) | Detachment | Specificity=4, Fit=1 | (--) |
|  |  |  |  | Excitement seeking (DAPP) | Disinhibited Externalizing | Specificity=4, Fit=0 | *At 4-month follow-up but not end of treatment:* |
|  |  |  |  |  |  |  | **Higher level:** ICAT outperformed CBT in reducing bulimic behavior and eating disorder psychopathology |
|  |  |  |  |  |  |  | **Lower level:** CBT outperformed ICAT n reducing bulimic behavior and eating disorder psychopathology |
|  |  |  |  | Self-control (SASB Intrex) | Disinhibited Externalizing | Specificity=4, Fit=0 | (--) |
|  |  |  |  | Depression (BDI) | Internalizing | Specificity=3, Fit=1 | (--) |
|  |  |  |  | Emotional lability (DAPP) | Internalizing | Specificity=4, Fit=0 | *At 4-month follow-up but not end of treatment:* |
|  |  |  |  |  |  |  | **Higher level:** ICAT outperformed CBT n reducing bulimic behavior and eating disorder psychopathology |
|  |  |  |  |  |  |  | **Lower level:** no significant difference n reducing bulimic behavior and eating disorder psychopathology |
|  |  |  |  | Dietary restraint (EDE) | Internalizing | Specificity=4, Fit=0 | (--) |
|  |  |  |  | Weight/shape concerns (EDE) | Internalizing | Specificity=4, Fit=0 | (--) |
|  |  |  |  | Anxiousness (STAI) | Internalizing | Specificity=4, Fit=1 | (--) |
|  |  |  |  | Self-blame (SASB Intrex) | Internalizing | Specificity=4, Fit=1 | (--) |
| Anderson et al., 2020 | Adults with BED, N=112, Mage=39.7, F=82, Non-White=9, Country=United States, Design=RCT | 1) Guided self-help CBT (CBTgsh), 2) Integrative Cognitive-Affective Therapy (ICAT) | EDE | Negative urgency (UPPS) | Disinhibited Externalizing | Specificity=4, Fit=0 | (++++) |
|  |  |  |  | Self-control (low) (SASB Intrex) | Disinhibited Externalizing | Specificity=4, Fit=0 | *At end of treatment but not at 6-month follow-up:* |
|  |  |  |  |  |  |  | **Lower level:** no significant difference between treatments |
|  |  |  |  |  |  |  | **Higher level:** ICAT-BED outperformed CBTgsh greater reductions in objective bulimic episode frequency, post-treatment |
|  |  |  |  | Negative affectivity (PANAS-X) | Internalizing | Specificity=1, Fit=1 | (--) |
|  |  |  |  | Dietary restraint (EDE-v16) | Internalizing | Specificity=4, Fit=0 | (--) |
|  |  |  |  | Weight/shape concerns (EDE-v16) | Internalizing | Specificity=4, Fit=0 | *At end of treatment but not at 6-month follow-up:* |
|  |  |  |  |  |  |  | **Lower level:** greater reductions in objective binge-eating episode frequency in ICAT than CBTgsh |
|  |  |  |  |  |  |  | **Higher level:** no significant difference between treatments post-treatment |
|  |  |  |  | Self-blame (SASB Intrex) | Internalizing | Specificity=4, Fit=1 | (++++) |
| de Jong et al., 2020 | Adult patients with an eating disorder, N=143, Mage=27.54, F=95.8, Country=Netherlands, Design=RCT | 1) CBT-E, 2) CBT-based TAU | EDEQ | General interpersonal distress (IIP) | General Psychopathology | Specificity=0, Fit=0 | (--) |
|  |  |  |  | Ineptitude (RSE) | Internalizing | Specificity=4, Fit=1 | **Higher level:** CBT-E outperformed CBT-based TAU between baseline and 6 weeks (no moderating effect on 20, 40, and 80 weeks from baseline) |
|  |  |  |  | Perfectionism (low) (FMPS) | Disinhibited Externalizing | Specificity=4, Fit=0 | (--) |
| Grilo et al., 2021 | Adults with binge-eating disorder N=457, Mage=36.6, F=36.8, Country=Australia, Design=RCT | 1) CBT, 2) Guided self-help CBT | Binge-eating remission (no binge-eating episodes/ past month) | Eating pathology (EDE) | Internalizing | Specificity=2, Fit=0 | (--) |
|  |  |  |  | Dietary restraint (EDE) | Internalizing | Specificity=4, Fit=0 | (--) |
|  |  |  |  | Weight concerns (EDE) | Internalizing | Specificity=4, Fit=0 | Significant interaction **CBT:** 43.5% with high Weight Concern and 61.0% with low Weight Concern remitted **Guided self-help CBT:** 44.1% of those with low Weight Concern compared to 56.3% with high Weight Concern remitted |
|  |  |  |  | Shape concerns (EDE) | Internalizing | Specificity=4, Fit=0 | (--) |
|  |  |  |  | Depression (BDI, HAM-D) | Internalizing | Specificity=3, Fit=1 | (--) |
| Gomez Penedo et al., 2019 | Women with DSM-III-R Bulimia Nervosa, N=220, Mage=28.1, F=100, Non-White=23, Country=United States, Design=RCT | 1) CBT, 2) IPT | Clinically meaningful improvement in global eating disorder symptomatology (EDE), 1-week purge frequency | Domineering (IIP-C) | Antagonistic Externalizing | Specificity=4, Fit=0 | **Higher level:** IPT outperformed CBT in symptom clinically meaningful improvement |
|  |  |  |  |  |  |  | **Lower level:** CBT outperformed IPT in symptom clinically meaningful improvement |
|  |  |  |  | Disaffiliativeness (Cold/distant - IIP-C) | Detachment | Specificity=4, Fit=0 | **Higher level:** no difference |
|  |  |  |  |  |  |  | **Lower level:** CBT outperformed IPT at likelihood of purging posttreatment |
| Lavender et al., 2012 | Patients with DSM- IV criteria for Bulimia Nervosa or Eating disorder not otherwise specified, N=74, Mage=27.7, F=92.5, Non-White=13, Country=United Kingdom, Design=RCT | 1) Group CBT, 2) Group Emotional and Social Mind Training | Eating disorder symptoms (EDE) | Avoidance (DTS) | Internalizing | Specificity=4, Fit=1 | (--) |
|  |  |  |  | Ineptitude (LSCS) | Internalizing | Specificity=4, Fit=1 | (--) |
|  |  |  |  | Submissiveness (SBS) | Internalizing | Specificity=4, Fit=1 | (--) |
| Le Grange et al., 2014 | Adults with DSM-IV Anorexia Nervosa, N=63, Mage=33.4, F=100, Country=Multiple countries, Design=RCT | 1) CBT, 2) Specialist supportive clinical management | Mental health (MCS), Depressive symptoms (BDI-II), eating disorder quality of life (EDQOL) | Eating pathology (EDE) | Internalizing | Specificity=2, Fit=0 | **Higher level:** CBT outperformed specialist supportive clinical management on MCS (post-treatment only) and BDI-II at 12-month follow-up not post-treatment or 6-month follow-up |
|  |  |  |  |  |  |  |  |
|  |  |  |  | Depression (BDI-II) | Internalizing | Specificity=3, Fit=1 | **Higher level:** CBT outperformed specialist supportive clinical management on MCS post-treatment only |
| Masheb & Grilo, 2008 | Adult patients who met DSM-IV research criteria for BED, N=75, Mage=46, F=81, Non-White=27, Country=United States, Design=RCT | 1) Guided self-help CBT, 2) Guided self-help Behavioral Weight Loss | Daily self-monitoring records, EDE-Q, BDI, BMI index | Distress (EDE-Q, TFEQ , BDI, RSES) | Internalizing | Specificity=2, Fit=1 | (--) |
| Robinson & Safer, 2012 | Adults with DSM-IV BED, N=101, Mage=52.2, F=85, Non-White=24, Country=United States, Design=RCT | 1) Group DBT, 2) Group Person-centered Counseling | Frequency of binge eating (EDE) | Eating pathology (EDE) | Internalizing | Specificity=2, Fit=0 | (--) |
|  |  |  |  | Depression (BDI) | Internalizing | Specificity=3, Fit=1 | (--) |
|  |  |  |  | Dietary restraint (EDE) | Internalizing | Specificity=4, Fit=0 | (--) |
|  |  |  |  | Shape concerns (EDE) | Internalizing | Specificity=4, Fit=0 | (--) |
|  |  |  |  | Weight concerns (EDE) | Internalizing | Specificity=4, Fit=0 | (--) |
|  |  |  |  | Ineptitude (RSE) | Internalizing | Specificity=4, Fit=1 | (--) |
| Schlup et al., 2010 | Female patients with BED, N=76, Mage=44.5, F=100, Country=Switzerland, Design=Non-RCT | 1) Standard CBT, 2) Brief CBT | Number of objective binges, dietary restraint, eating concern, weight concern, shape concern (EDE) | Distress (BDI, BAI, EDE) | Internalizing | Specificity=2, Fit=1 | Significant interaction over 6 and 12-month follow-up |
|  |  |  |  |  |  |  | **Brief CBT:** higher level (based on cluster analysis derived subtype) higher EDE eating concern than those of lower level |
|  |  |  |  |  |  |  | **Standard CBT:** similar |
| Sysko et al., 2010 | Patients with DSM-IV BED, N=205, Mage=48.5, F=85.36, Non-White=18, Country=United States, Design=RCT | 1) Guided self-help CBT, 2) IPT, 3) Behavioral Weight Loss | Objective/ subjective bulimic episodes, BMI, Weight and Shape Concern, BDI, Dietary Restraint | Depression (BDI) | Internalizing | Specificity=3, Fit=1 | **Higher level (latent class derived from individual moderators):** IPT outperformed CBT in the probability of remission from binge eating |
|  |  |  |  | Dietary restraint (EDE-Q) | Internalizing | Specificity=4, Fit=0 |  |
|  |  |  |  | Shape concerns (EDE-Q) | Internalizing | Specificity=4, Fit=0 |  |
|  |  |  |  | Weight concerns (EDE-Q) | Internalizing | Specificity=4, Fit=0 |  |
| Tasca et al., 2006 | Patients with BED, N=135, Mage=42.75, F=91.11, Non-White=2.30, Country=Canada, Design=RCT | 1) Group CBT, 2) Group Psychodynamic Interpersonal Psychotherapy, 3) Waitlist | Days binged | Intimacy Avoidance (ASQ) | Detachment | Specificity=4, Fit=1 | (--) |
|  |  |  |  | Need for approval (ASQ) | Internalizing | Specificity=4, Fit=1 | Significant interaction |
|  |  |  |  |  |  |  | **Higher level:** group psychodynamic interpersonal psychotherapy outperformed CBT at 12-month follow-up |
|  |  |  |  |  |  |  | **Lower level:** CBT outperformed psychodynamic interpersonal psychotherapy at 12-month follow-up |
|  |  |  |  | Separation insecurity (ASQ) | Internalizing | Specificity=4, Fit=0 | (--) |
| Wade et al., 2021 | Adults with an eating disorder, N=98, Mage=26.31, F=45.71, Non-White=17.66, Country=Australia, Design=RCT | 1) Brief CBT with content on body image, 2) Brief CBT with motivation enhancement | Global Eating Disorder Psychopathology (EDE-Q) | Body avoidance (BIAQ) | Internalizing | Specificity=4, Fit=0 | (--) |
|  |  |  |  | Body checking (BCQ) | Internalizing | Specificity=4, Fit=0 | (--) |
| Wilson et al., 2010 | Patients with DSM-IV BED, N=205, Mage=48.4, F=85.33, Non-White=17.66, Country=United States, Design=RCT | 1) Guided self-help CBT, 2) IPT, 3) Behavioral Weight Loss | Binge eating remission (EDE) | Eating pathology (EDE) | Internalizing | Specificity=2, Fit=0 | **Higher level**: IPT outperformed ORs between low and high levels of the EDE were: BWL, 2.8; CBTgsh, 2.9; and IPT, 0.7 at 2-year follow-up |
|  |  |  |  | Depression (BDI) | Internalizing | Specificity=3, Fit=1 | (--) |
|  |  |  |  | Ineptitude (RSE) | Internalizing | Specificity=4, Fit=1 | **Lower level:** IPT outperformed ORs between low and high levels of the EDE were: 2.4 for BWL, 1.9 for CBTgsh, and 0.9 for IPT at 2-year follow-up |

**B.1.3. Treatment for fear disorders**

| **Study** | **Study Characteristics** | **Treatments** | **Outcome** | **Moderator** | **Spectrum** | **Moderator Characteristics** | **Result** |
| --- | --- | --- | --- | --- | --- | --- | --- |
| Borge et al., 2010 | Patients with Social Phobia, N=80, Mage=37.5, F=51.3, Country=United States, Design=RCT | 1) CT, 2) IPT | Social phobia symptoms (SPAI) | General interpersonal distress (IIP-64) | General Psychopathology | Specificity=0, Fit=0 | (++++) |
|  |  |  |  | Depression (BDI-II) | Internalizing | Specificity=3, Fit=1 | (--) |
|  |  |  |  | Avoidant PD (SCID-II interview) | Detachment | Specificity=3, Fit=1 | (--) |
| Chambless et al., 2017 | Adults with Panic Disorder, N=161, Mage=39.4, F=64.6, Non-White=26.7, Country=United States, Design=RCT | 1) CBT, 2) Panic-Focused Psychodynamic Psychotherapy | Panic Disorder severity (PDSS) | Agoraphobic avoidance (MIA) | Internalizing | Specificity=4, Fit=1 | (--) |
| Cludius et al., 2020 | Patients with OCD, non-responders to CBT, N=125, Mage=38.62, F=61, Country=Germany, Design=RCT | 1) Group MBCT, 2) Group Psychoeducation | OCD symptoms | OCD (Y-BOCS) | Internalizing | Specificity=3, Fit=0 | (--) |
|  |  |  | Obsessions, |  |  |  |  |
|  |  |  | Compulsions, (Y-BOCS) |  |  |  |  |
| Craske et al., 2014 | Adults with DSM-IV principal or co-principal social phobia, generalized type, N=87, Mage=28.37, F=45.98, Country=United States, Design=RCT | 1) CBT, 2) ACT, 3) Waitlist | Social phobia symptom severity (LSAS–SR, SIAS, SPS), fear and avoidance severity (ADIS–IV CSR) | Extraversion (EPQ) | Detachment | Specificity=1, Fit=1 | (--) |
|  |  |  |  | Control over anxiety (ACQ) | Internalizing | Specificity=4, Fit=1 | (--) |
|  |  |  |  | Avoidance (AAQ) | Internalizing | Specificity=4, Fit=1 | **Higher level:** CBT outperformed ACT in social phobia symptom reduction by 12-month follow-up |
|  |  |  |  | Neuroticism (EPQ) | Internalizing | Specificity=1, Fit=1 | (--) |
| Dow et al., 2007 | Patients with Panic Disorder, N=72, Country=Multiple countries, Design=RCT | 1) CBT, 2) Brief Individual CBT | Composite panic/anxiety score | Agoraphobia (FQ) | Internalizing | Specificity=3, Fit=1 | (--) |
|  |  |  |  | Anxiousness (STAI) | Internalizing | Specificity=3, Fit=1 | Moderated outcome only when pretreatment panic symptoms were not controlled in the model -not probed |
|  |  |  |  | Depression (BDI) | Internalizing | Specificity=3, Fit=1 | **Higher level:** Standard CBT outperformed brief CBT post-treatment |
|  |  |  |  | Fear of Blood/injury (FQ) | Internalizing | Specificity=3, Fit=1 | (--) |
|  |  |  |  | Panic disorder | Internalizing | Specificity=4, Fit=1 | (--) |
|  |  |  |  | Social anxiety (FQ) | Internalizing | Specificity=4, Fit=1 | (--) |
| El Alaoui et al., 2013 | Adults with Panic Disorder, N=104, Mage=34.2, F=62, Country=Sweden, Design=RCT | 1) Internet guided CBT, 2) Group CBT | Panic disorder severity (PDSS) | Panic disorder (PDSS) | Internalizing | Specificity=3, Fit=1 | (--) |
|  |  |  |  | Depression (MADRS) | Internalizing | Specificity=3, Fit=1 | (--) |
|  |  |  |  | Anxiety sensitivity (ASI) | Internalizing | Specificity=4, Fit=1 | (++++) |
| Hedman et al., 2012 | Patients with social anxiety disorder, N=126, Mage=35.35, F=35.71, Country=Sweden, Design=RCT | 1) Internet-based CBT, 2) Group CBT | Social anxiety severity (LSAS), SAD diagnosis (SCID-I RV) | Excitement seeking (SSP) | Disinhibited Externalizing | Specificity=4, Fit=0 | (++++) |
|  |  |  |  | Impulsivity (SSP) | Disinhibited Externalizing | Specificity=4, Fit=0 | (++++) |
|  |  |  |  | Anxiety (BAI) | Internalizing | Specificity=3, Fit=1 | **Internet-based CBT:** higher levels worse outcomes than lower levels |
|  |  |  |  |  |  |  | **Group CBT:** no difference across levels |
|  |  |  |  | Social anxiety (LSAS) | Internalizing | Specificity=3, Fit=1 | (--) |
|  |  |  |  | Depression (MADRS-S) | Internalizing | Specificity=3, Fit=1 | (--) |
|  |  |  |  | Anxiety sensitivity | Internalizing | Specificity=4, Fit=1 | (--) |
| Hunt et al., 2006 | Undergraduates with a self-reported fear of snakes, N=60, Mage=22, F=71.67, Country=United States, Design=RCT | 1) Cognitive Restructuring, 2) In vivo Exposure | Snake phobia symptoms (SQ), Level of discomfort in a behavioral approach test (BAT) | Snake phobia (SQ) | Internalizing | Specificity=3, Fit=1 | **Higher level:** cognitive imagery modification outperformed vivo exposure post intervention |
| Maher et al., 2010 | Participants with DSM-IV diagnosis of OCD receiving serotonin reuptake inhibitor, N=108, Mage=39.2, F=42.6, Country=United States, Design=RCT | 1) CBT-Exposure and Response Prevention (EX/RP), 2) CBT-Stress management training (SMT) | OCD symptoms (YBOCS) | Anxiety (HAM-A) | Internalizing | Specificity=3, Fit=1 | (--) |
|  |  |  |  | Depression (HAM-D) | Internalizing | Specificity=3, Fit=1 | (--) |
|  |  |  |  | OCD (YBOCS) | Internalizing | Specificity=3, Fit=0 | **EX/RP:** no difference across levels post-treatment |
|  |  |  |  |  |  |  | **SMT:** poorer outcome among at higher level post-treatment |
| Mesri et al., 2017 | Participants with DSM-IV principal or co-principal social phobia, generalized type, N=49, Mage=28.46, F=42.31, Non-White=46.15, Country=United States, Design=RCT | 1) CBT, 2) ACT | Stress reactivity (STAI), Social anxiety symptoms (LSAS–SR, SIAS, SPS), Quality of life (QoLIa) | Public speaking avoidance (ADIS‐IV CSR) | Internalizing | Specificity=4, Fit=1 | (--) |
|  |  |  |  | Public speaking fear (ADIS‐IV CSR) | Internalizing | Specificity=4, Fit=1 | **Lower level**: higher quality of life in ACT than CBT by 12-month follow-up |
|  |  |  |  |  |  |  | **Higher level:** non-significant trend for higher quality of life in CBT than ACT by 12-month follow-up |
|  |  |  |  |  |  |  | (only at 6-month follow-up not post-treatment or 12-month follow-up) |
| Mesri et al., 2020 | Individuals with panic disorder with or without agoraphobia, N=66, Mage=35.13, F=59.09, Non-White=30.31, Country=United States, Design=RCT | 1) CBT, 2) CBT-In Vivo Exposures | Agoraphobia (ADIS–IV CSR) | Agoraphobic avoidance (ADIS‐IV CSR) | Internalizing | Specificity=4, Fit=1 | (--) |
|  |  |  |  | Agoraphobic fear (ADIS‐IV CSR) | Internalizing | Specificity=4, Fit=1 | (--) |
| Sewart et al., 2019 | Adults with either principal or co-principal social phobia, generalized type, N=111, Mage=28.3, F=50, Non-White=53.2, Country=United States, Design=RCT | 1) CBT, 2) ACT, 3) Waitlist | Social anxiety symptoms (composite of LSAS–SR, SIAS, SPS) | Negative affectivity (PANAS) | Internalizing | Specificity=1, Fit=1 | (--) |
|  |  |  |  | Positive affect (low) (PANAS) | Internalizing | Specificity=4, Fit=0 | (++++) |
| Stapinski et al., 2020 | Adults with co-occurring SAD and AUD symptoms, N=117, Mage=36.6, F=36.8, Country=Australia, Design=RCT | 1) AUD-focused CBT + Motivational Enhancement Therapy, 2) Integrated CBT + Motivational Enhancement Therapy | Alcohol dependence severity (SADQ), Social Anxiety (ADIS–IV CSR, SPS, SIAS), Quality of life (QoL) | AUD (SADQ) | Disinhibited Externalizing | Specificity=3, Fit=1 | **Higher level:** Integrated treatment outperformed alcohol-focused treatment at 3-month (not 6-month) follow-up only in the alcohol dependence outcome **Lower level:** no difference |
| Steketee et al., 2019 | Outpatients diagnosed with OCD, N=359, Mage=35.93, F=60, Country=Multiple countries, Design=Mixed | 1) CBT, 2) CT, 3) Behavior Therapy | Reliable change in OCD symptoms (Y-BOCS) | Depression (BDI) | Internalizing | Specificity=3, Fit=1 | **Behavior therapy:** higher levels worse treatment outcomes than low levels |
|  |  |  |  |  |  |  | **CBT and CT:** no difference across levels |
|  |  |  |  | OCD (Y-BOCS) | Internalizing | Specificity=3, Fit=0 | **CT:** higher levels predicted worse outcomes |
|  |  |  |  |  |  |  | **CBT and Behavior therapy:** no difference across levels |
| Strauss et al., 2017 | Patients with social anxiety disorder, N=412, Mage=35.4, F=57, Country=Germany, Design=RCT | 1) CBT, 2) Brief Individual Psychodynamic Psychotherapy | Social anxiety symptoms (LSAS) | Intimacy Avoidance (ECR-R) | Detachment | Specificity=4, Fit=1 | (--) |
|  |  |  |  | Social anxiety (LSAS) | Internalizing | Specificity=3, Fit=1 | Significant moderation at 1-year follow-up |
|  |  |  |  |  |  |  | Interaction not probed |
|  |  |  |  | Separation insecurity (ECR-R) | Internalizing | Specificity=4, Fit=0 | (--) |
| Vos, Huibers, & Arntz, 2012 | Adults with a primary DSM-III/DSM-IV diagnosis of OCD with overt compulsions, N=78, Mage=30.62, F=62.82, Country=Netherlands, Design=RCT | 1) CT-R, 2) CT-Overestimations of Danger | Change in OCD symptoms (PI-R, MOCI), phobia ratings (FQ) | Checking (PIR) | Internalizing | Specificity=4, Fit=0 | (--) |
|  |  |  |  | Compulsions (PIR) | Internalizing | Specificity=4, Fit=0 | (--) |
|  |  |  |  | Obsessive thoughts (PIR) | Internalizing | Specificity=4, Fit=0 | (--) |
|  |  |  |  | Precision (PIR) | Internalizing | Specificity=4, Fit=0 | (--) |
|  |  |  |  | Washing (PIR) | Internalizing | Specificity=4, Fit=0 | (--) |

**B.1.4. Treatment for one or more internalizing disorders**

| **Study** | **Study Characteristics** | **Treatments** | **Outcome** | **Moderator** | **Spectrum** | **Moderator characteristics** | **Result** |
| --- | --- | --- | --- | --- | --- | --- | --- |
| Arch et al., 2013 | Veterans with a DSM-IV anxiety disorder, N=71, Mage=46.6, F=20.6, Non-White=31.3, Country=United States, Design=RCT | 1) Group CBT, 2) Group mindfulness-based stress reduction (MBSR) | CSR-MINI | Depression (BDI-II) | Internalizing | Specificity=3, Fit=1 | **Lower level:** CBT outperformed MBSR at follow-up |
|  |  |  |  |  |  |  | **Moderate to severe:** MBSR outperformed CBT at follow-up |
|  |  |  |  | Anxiety sensitivity (ASI) | Internalizing | Specificity=4, Fit=1 | **High level:** CBT outperformed MBSR at end of treatment only |
|  |  |  |  |  |  |  | **Average level:** MBSR outperformed CBT at follow-up |
| Donker et al., 2013 | Individuals with depression and anxiety symptoms, N=1,843, Age=59% < 40, F=72.4, Country=Australia, Design=RCT | 1) Internet unguided CBT, 2) Internet unguided IPT, 3) Internet unguided control CBT (MoodGYM) | Depression severity (CES-D) | Depression (CES-D) | Internalizing | Specificity=3, Fit=1 | (--) |
|  |  |  |  | Generalized anxiety (GAD-7) | Internalizing | Specificity=4, Fit=1 | (--) |
| Eskildsen et al., 2020 | Adults with MDD, social anxiety disorder, agoraphobia, and/or panic disorder, N=291, Country=Denmark, Design=RCT | 1) Disorder-specific group CBT, 2) Transdiagnostic group CBT | Well-being (WHO-5 Well-being Index) | Antagonism (PID-5) | Antagonistic Externalizing | Specificity=1, Fit=1 | (--) |
|  |  |  |  | Detachment (PID-5) | Detachment | Specificity=1, Fit=1 | (--) |
|  |  |  |  | Disinhibition (PID-5) | Disinhibited Externalizing | Specificity=1, Fit=1 | (--) |
|  |  |  |  | Negative affectivity (PID-5) | Internalizing | Specificity=1, Fit=1 | (--) |
|  |  |  |  | Anxiety (HAM-A6) | Internalizing | Specificity=3, Fit=1 | (--) |
|  |  |  |  | Depression (BDI-II) | Internalizing | Specificity=3, Fit=1 | (--) |
|  |  |  |  | Positive affect (low) (PANAS) | Internalizing | Specificity=4, Fit=0 | (--) |
|  |  |  |  | Psychoticism (PID-5) | Thought Disorder | Specificity=1, Fit=1 | (--) |
| Johnson & Hoffart, 2019 | Inpatients with a primary diagnosis of PTSD, social phobia, or panic disorder with and without agoraphobia, N=74, Mage=42, F=60.81, Country=Norway, Design=RCT | 1) CBT, 2) Metacognitive Therapy | Generalized anxiety symptoms (BAI) | Avoidance (ADIS‐IV DSM‐IV) | Internalizing | Specificity=4, Fit=1 | (--) |
|  |  |  |  | Worry (PSWQ) | Internalizing | Specificity=4, Fit=1 | (--) |
| Mars et al., 2020 | Adults who accessed high-intensity community-based psychological treatment for depression and/or anxiety disorder, N=3,689, Mage=44.5, F=71.7, Country=United Kingdom, Design=Retrospective | 1) CBT, 2) Emotional Skills Training based on DBT | Recovery from and reliable improvement in depression (PHQ-9), Recovery from and reliable improvement in anxiety (GAD-7) | General Psychopathology (General personality pathology - SAPAS) | General Psychopathology | Specificity=0, Fit=1 | (--) |
|  |  |  |  | Depression (BDI-II) | Internalizing | Specificity=3, Fit=1 | (--) |
| Proudfoot et al., 2003 | Adults suffering from anxiety and/or depression, N=167, Mage=44.7, F=73.65, Non-White=12, Country=United Kingdom, Design=RCT | 1) Internet unguided CBT, 2) TAU | Depression severity (BDI), Work and Social Adjustment (WSAS), Anxiety (BAI) | Depression (BDI) | Internalizing | Specificity=3, Fit=1 | (--) |
| Proudfoot et al., 2004 | General practice patients suffering from anxiety and/or depression, N=274, Mage=43.51, F=73.72, Non-White=19.71, Country=United Kingdom, Design=RCT | 1) Internet unguided CBT (Beating the Blues), 2) TAU | Anxiety (BAI), Depression severity (BDI-II) | Anxiety (BAI) | Internalizing | Specificity=3, Fit=1 | Significant interaction at 6-month follow-up  **Higher level:** CBT outperformed TAU in anxiety reduction  **Lower level**: no difference between conditions |
|  |  |  |  | Depression (BDI-II) | Internalizing | Specificity=3, Fit=1 | (--) |
| Schwartz et al., 2021 | Adults with any Internalizing Disorder N=1,379, F=68.31, Non-White=19.71, Country=Germany, Design=Naturalistic study | 1) CBT, 2) Psychodynamic Psychotherapy | General psychopathology (BSI) | General psychopathology (BSI) | General Psychopathology | Specificity=0, Fit=1 | (--) |
|  |  |  |  | Psychoticism (BSI) | Thought Disorder | Specificity=1, Fit=1 | (--) |
|  |  |  |  | Somatization (BSI) | Somatoform | Specificity=4, Fit=0 | (--) |
|  |  |  |  | Ineptitude (BSI) | Internalizing | Specificity=4, Fit=1 | (--) |
|  |  |  |  | Depression (BSI) | Internalizing | Specificity=3, Fit=1 | Unclear relationship in the multivariable model predicting differential treatment response |
|  |  |  |  | Anxiety (BSI) | Internalizing | Specificity=3, Fit=1 | (--) |
|  |  |  |  | Hostility (BSI) | Internalizing | Specificity=4, Fit=1 | (--) |
|  |  |  |  | Behavior restricting fears (BSI) | Internalizing | Specificity=4, Fit=1 | Unclear relationship in the multivariable model predicting differential treatment response |
|  |  |  |  | Paranoid ideation (BSI) | Thought Disorder | Specificity=4, Fit=1 | Unclear relationship in the multivariable model predicting differential treatment response |
|  |  |  |  | Depression (BDI-II) | Internalizing | Specificity=3, Fit=1 | (--) |
|  |  |  |  | Dominance (IIP-D) | Antagonistic Externalizing | Specificity=4, Fit=0 | (--) |
|  |  |  |  | Submissiveness (IIP-D) | Internalizing | Specificity=4, Fit=0 | (--) |
| Wolitzky-Taylor et al., 2012 | Patients with DSM-IV diagnostic criteria for one or more anxiety disorders, N=87, Mage=37.93, F=47.1, Non-White=33.3, Country=United States, Design=RCT | 1) CBT, 2) ACT | Anxiety (MASQ-GA) | Neuroticism (NEO-PI-R) | Internalizing | Specificity=1, Fit=1 | (--) |
|  |  |  |  | Anxiety sensitivity (ASI) | Internalizing | Specificity=4, Fit=1 | Significant non-linear moderation |
|  |  |  |  |  |  |  | **Moderate level:** CBT outperformed ACT across post-treatment, and 6- and 12-month follow-up |
|  |  |  |  | Avoidance (AAQ-16) | Internalizing | Specificity=4, Fit=1 | Significant non-linear moderation |
|  |  |  |  |  |  |  | Probing the interaction did not show significant differences in the effectiveness of treatment conditions across post-treatment, and 6- and 12-month follow-up |

*Note*: ASI = Anxiety Sensitivity Index; AAQ = Acceptance and Action Questionnaire–16; ACQ = Anxiety Control Questionnaire; ADIS-IV = Anxiety Disorders Interview Schedule; ASQ = Attachment Styles Questionnaire; B-YAACQ = Brief Young Adult Alcohol Consequences Questionnaire; BAI = Beck Anxiety Inventory; BAS = Behavioral Activation System Scale; BAT = Behavioral Approach Test; BDI = Beck Depression Inventory; BFI = ‘Big Five’ Inventory; BHS = Beck Hopelessness Scale; BSI = Brief Inventory of Symptoms; BSS-I = Beck Scale for Suicide Ideation; CBAS = Cognitive-Behavioral Avoidance Scale; CES-D = Center for Epidemiological Studies Depression; CIS-R = Clinical Interview Schedule—Revised form; CIWAS = Clinical Institute Withdrawal from Alcohol Scale; CSR = Clinician severity rating; D5D = Système de Description en Cinq dimensions; DAPP = Dimensional Assessment of Personality Pathology; DAS = Dysfunctional Attitude Scale; DASS = Depression Anxiety Stress Scales; DEQ = Depressive Experiences Questionnaire; ECR-R = Experiences in Close Relation- ships Scale; EDE = Eating Disorder Examination Questionnaire; EDQOL = Eating Disorders Quality of Life; EPQ = Eysenck Personality Questionnaire; FDAS = Four Dimensional Anxiety Scale; FMPS = Frost Multidimensional Perfectionism Scale; FQ = Fear Questionnaire; GAD-7 = Generalized Anxiety Disorder-7; GAF = Global Assessment of Functioning scale; HADS-A = Hospital Anxiety and Depression Scale - Anxiety subscale ; HAM-A = Hamilton Anxiety Rating Scale ; HDRS = Hamilton Depression Rating Scale; IIP = Inventory of Interpersonal Problems; IPDE = International Personality Disorder Examination; ISI = Insomnia Severity Index; LSAS = Liebowitz Social Anxiety Scale; LSCS = Levels of Self-Criticism Scale; MADRS = Montgomery-Asberg Depression Rating Scale; MCS = Mental Component Summary scale score of the Short Form-12 Health Survey; MDI = Trauma-specific dissociation scale of the Multiscale Dissociation Inventory; MHC-SF = Mental Health Continuum- Short Form; MIA = Mobility Inventory for Agoraphobia; NEO = Revised NEO Personality Inventory; NVM = Shortened Dutch Adaptation of the Minnesota Multiphasic Personality Inventory; OTI = Opiate Treatment Index; PANAS = Positive and Negative Affect Schedule; PANAS-X = Positive and Negative Affect Schedule—Expanded Version; PDS = Posttraumatic Diagnostic Scale; PDSS = Panic Disorder Severity Scale; PHQ-9 = Patient Health Questionnaire ; PID-5 = The Personality Inventory for DSM-5 ; PIR = Padua Inventory Revised; PSS-I = PTSD Symptom Scale-Interview; PSWQ = Penn State Worry Questionnaire; PWBS = Psychological Well-Being Scales; QoLI = Quality of Life Inventory; RSE = Rosenberg Self-Esteem Scale; RSQ = Relationship Scales Questionnaire; SADQ = Severity of Alcohol Dependence Questionnaire; SAPAS = Standardized Assessment of Personality – Abbreviated Scale; SASB INTREX = Structural Analysis of Social Behavior INTREX Questionnaire; SBS = Submissive Behaviour Scale; SCID-II = Structured Clinical Interview for DSM-IV Axis II ; SCL-90 = Symptom Checklist-90; SCL-K-9 = Symptom-Checklist-K-9; SIAS = Social Interaction Anxiety Scale; SLSC-R = Self-Liking/Self Competence Scale; SPAI = Social Phobia and Anxiety Inventory; SPS = Social Phobia Scale; SPSI-R = Social Problem-Solving Inventory-Revised; SQ = Snake Questionnaire; SSP = Swedish Scales of Personality; STAI = Spielberger Trait Anxiety Inventory ; SWE = Self-Efficacy Scale; TLFB = The Alcohol Timeline Followback ; TSI= Dissociation Scale of the Trauma Symptom Inventory; UPPS = UPPS Impulsive Behavior Scale; WSAS = Work and Social Adjustment Scale; YBOCS = Yale-Brown Obsessive Compulsive Scale

## **Appendix C**

## **Table C.1**

*Moderators examined in the reviewed studies and their significance*

| **HiTOP dimension** | **Specificity** | **Distress Disorders** | **Eating Disorders** | **Fear Disorders** | **Combination of Internalizing Disorders** |
| --- | --- | --- | --- | --- | --- |
| ***General psychopathology*** |  |  |  |  |  |
| General psychopathology | Superspectrum | Yes | No | No | No |
|  |  |  |  |  |  |
| ***Antagonistic EXT*** |  |  |  |  |  |
| Agreeableness | Spectrum | No | - | - | - |
| Antagonism | Spectrum | - | - | - | No |
| Antagonistic Externalizing | Spectrum | No | - | - | - |
| Aggression | Symptom/trait | No | - | - | - |
| Dominance | Symptom/trait | Yes | Yes | - | - |
| Attention seeking | Symptom/trait | Yes | - | - | - |
| Vindictiveness | Symptom/trait | Yes | - | - | - |
|  |  |  |  |  |  |
| ***Detachment*** |  |  |  |  |  |
| Detachment | Spectrum | No | - | - | No |
| Extraversion | Spectrum | Yes | - | No | - |
| Avoidant PD | Syndrome | Yes | - | No | - |
| Intimacy Avoidance | Symptom/trait | No | No | No | - |
| Disaffilliativeness | Symptom/trait | No | Yes | - | - |
| Social Withdrawal | Symptom/trait | No | - | - | - |
|  |  |  |  |  |  |
| ***Disinhibited EXT*** |  |  |  |  |  |
| Conscientiousness | Spectrum | No | - | - | - |
| Disinhibition | Spectrum | - | - | - | No |
| Alcohol Use Disorder | Syndrome | - | - | Yes | - |
| Alcohol use | Symptom/trait | Yes | - | - | - |
| Alcohol problems | Symptom/trait | - | - | - | No |
| Drug problems | Symptom/trait | - | - | - | Yes |
| Excitement seeking | Symptom/trait | - | Yes | No | - |
| Impulsivity | Symptom/trait | No | - | No | - |
| Negative urgency | Symptom/trait | - | No | - | - |
| Perfectionism (low) | Symptom/trait | Yes | No | - | - |
| Risk taking | Symptom/trait | No | - | - | - |
|  |  |  |  |  |  |
| ***Internalizing*** |  |  |  |  |  |
| Negative affectivity/Neuroticism | Spectrum | No | No | No | No |
| Distress | Subfactor | - | Yes | - | - |
| Eating pathology | Subfactor | - | Yes | - | - |
| Fear | Subfactor | No | - | - | - |
| Agoraphobia | Syndrome | - | - | No | - |
| Anxiety | Syndrome | Yes | - | Yes | Yes |
| Depression | Syndrome | Yes | Yes | Yes | Yes |
| Obsessive PD | Syndrome | Yes | - | - | - |
| OCD | Syndrome | - | - | Yes | - |
| Panic disorder | Syndrome | - | - | Yes | - |
| PTSD | Syndrome | Yes | - | - | - |
| Social anxiety | Syndrome | - | - | Yes | - |
| Specific phobia | Syndrome | - | - | Yes | - |
| Activation | Symptom/trait | No | - | - | - |
| Anxiety sensitivity | Symptom/trait | Yes | - | Yes | Yes |
| Anxiousness | Symptom/trait | - | No | - | - |
| Avoidance | Symptom/trait | No | No | Yes | Yes |
| Behavior restricting fears | Symptom/trait | No | - | - | Yes |
| Checking | Symptom/trait | - | - | - | - |
| Compulsions | Symptom/trait | - | - | - | - |
| Dissociation | Symptom/trait | Yes | - | - | - |
| Dietary restraint | Symptom/trait | - | Yes | - | - |
| Emotional lability | Symptom/trait | - | Yes | - | - |
| Fear of Blood/injury | Symptom/trait | - | - | No | - |
| Generalized anxiety symptoms | Symptom/trait | No | - | - | No |
| Hopelessness | Symptom/trait | No | - | - | - |
| Hostility | Symptom/trait | Yes | - | - | - |
| Ineptitude | Symptom/trait | No | Yes | - | - |
| Insomnia | Symptom/trait | No | - | - | - |
| Need for approval | Symptom/trait | No | Yes | - | - |
| Pure obsessions | Symptom/trait | - | - | No | - |
| Positive affect (low) | Symptom/trait | No | - | No | No |
| Positive relations (low) | Symptom/trait | - | - | - | No |
| Rituals | Symptom/trait | - | - | No | - |
| Public speaking fear | Symptom/trait | - | - | Yes | - |
| Separation insecurity | Symptom/trait | No | No | No | - |
| Submissiveness | Symptom/trait | Yes | - | - | - |
| Suicidality | Symptom/trait | No | - | - | - |
| Cleaning | Symptom/trait | - | - | No | - |
| Weight/shape concerns | Symptom/trait | - | Yes | - | - |
| Well-being | Symptom/trait | No | - | - | - |
| Worry | Symptom/trait | - | - | - | No |
|  |  |  |  |  |  |
| ***Somatoform*** |  |  |  |  |  |
| Cognitive complaints | Symptom/trait | Yes | - | - | - |
| Somatization | Symptom/trait | Yes | - | - | - |
|  |  |  |  |  |  |
| ***Thought disorder*** |  |  |  |  |  |
| Openness | Spectrum | No | - | - | - |
| Psychoticism | Spectrum | - | - | - | - |
| Thought disorder | Spectrum | Yes | - | - | - |
| Paranoid ideation | Symptom/trait | Yes | - | - | Yes |

*Note*: Yes = was found significant at least once in this disorder class; No = was not found significant in this disorder class; EXT = externalizing

**Appendix D**

**Figure D.1**

*Hierarchical Taxonomy of Psychopathology (as proposed by Kotov et al., 2017)*


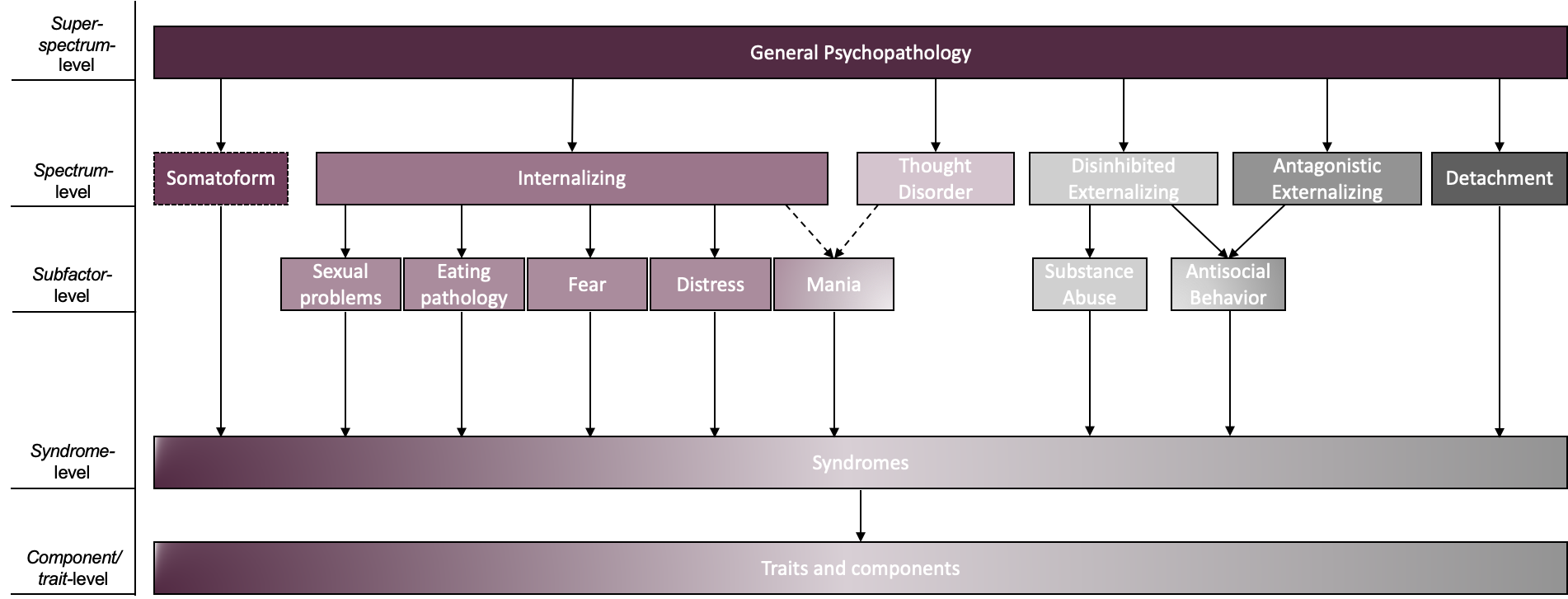


**Appendix E**

**Figure E.1**

*Example individualized decision rule for personalized treatment selection based on a set of HiTOP dimensions.*


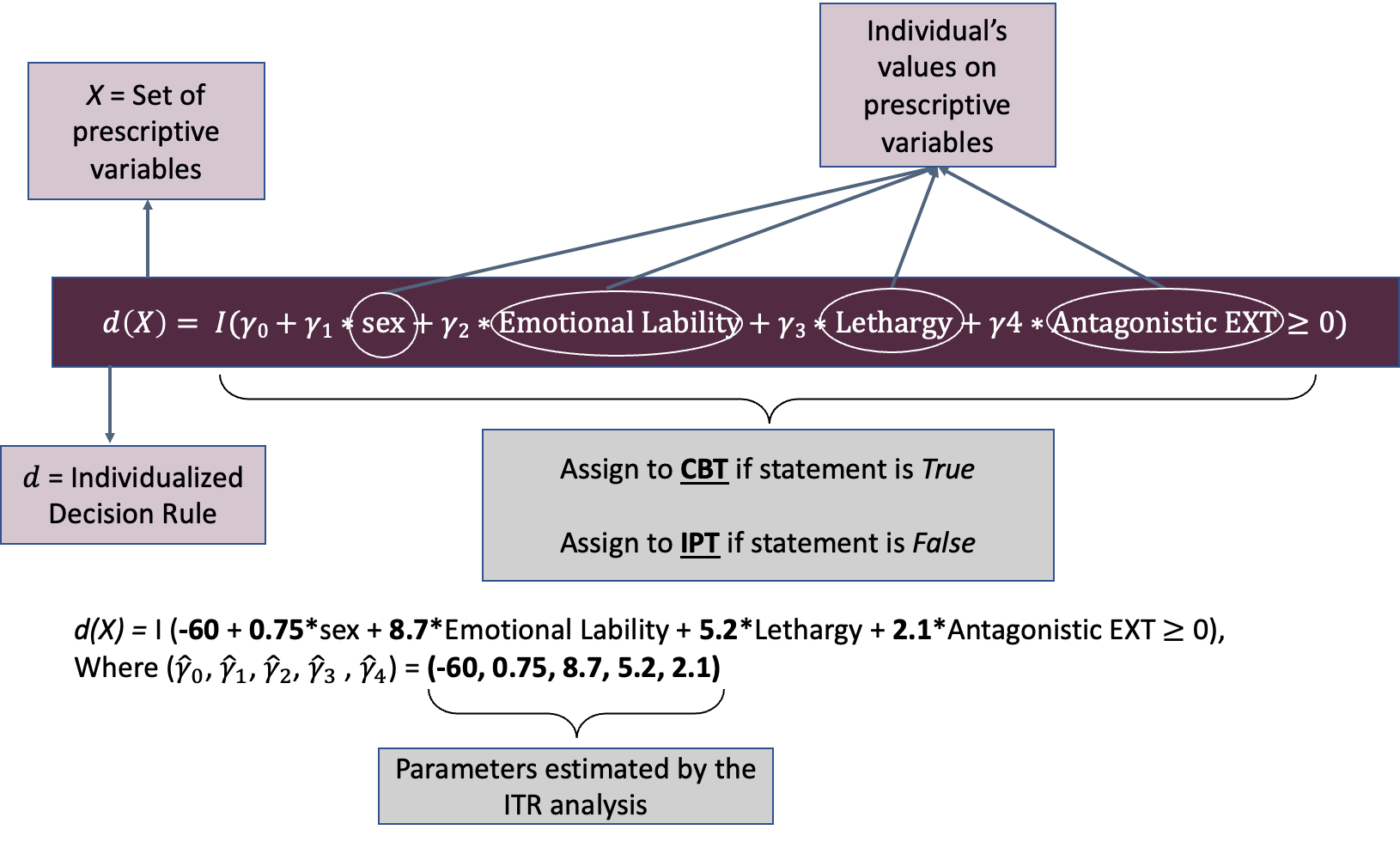


**Appendix F**

**Figure F.1**

*Flow chart of the study selection process with specific reasons for study exclusion*

**
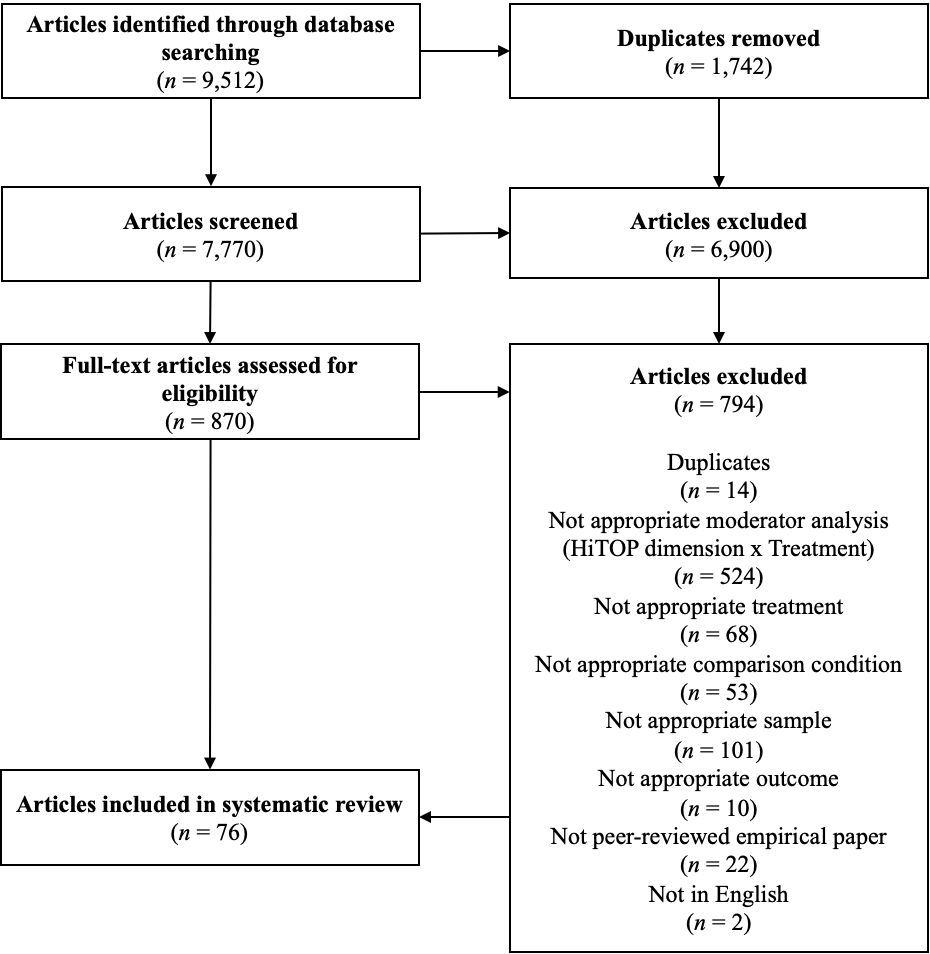
**

**Appendix G**

**Figure G.1**

*Heatmaps of % studies with moderating effect significance within spectra and across different specificity levels.*


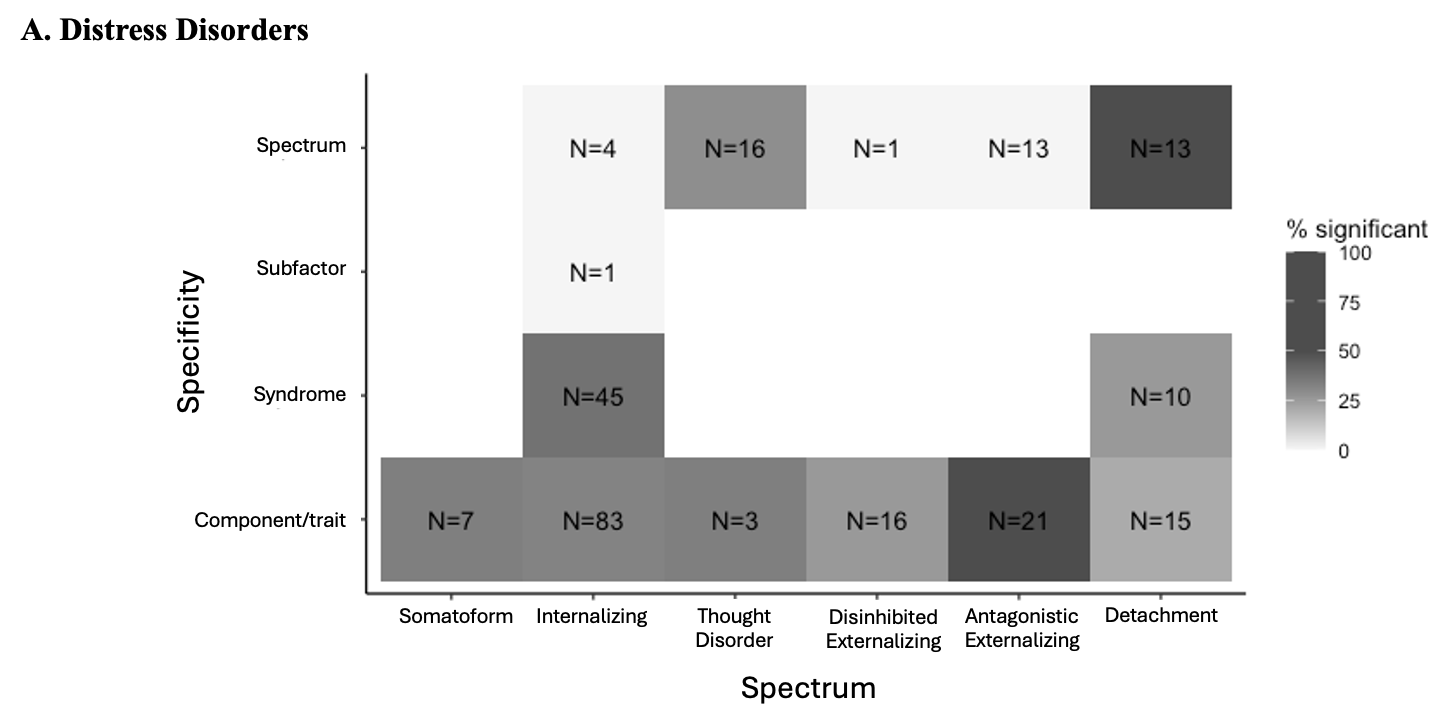


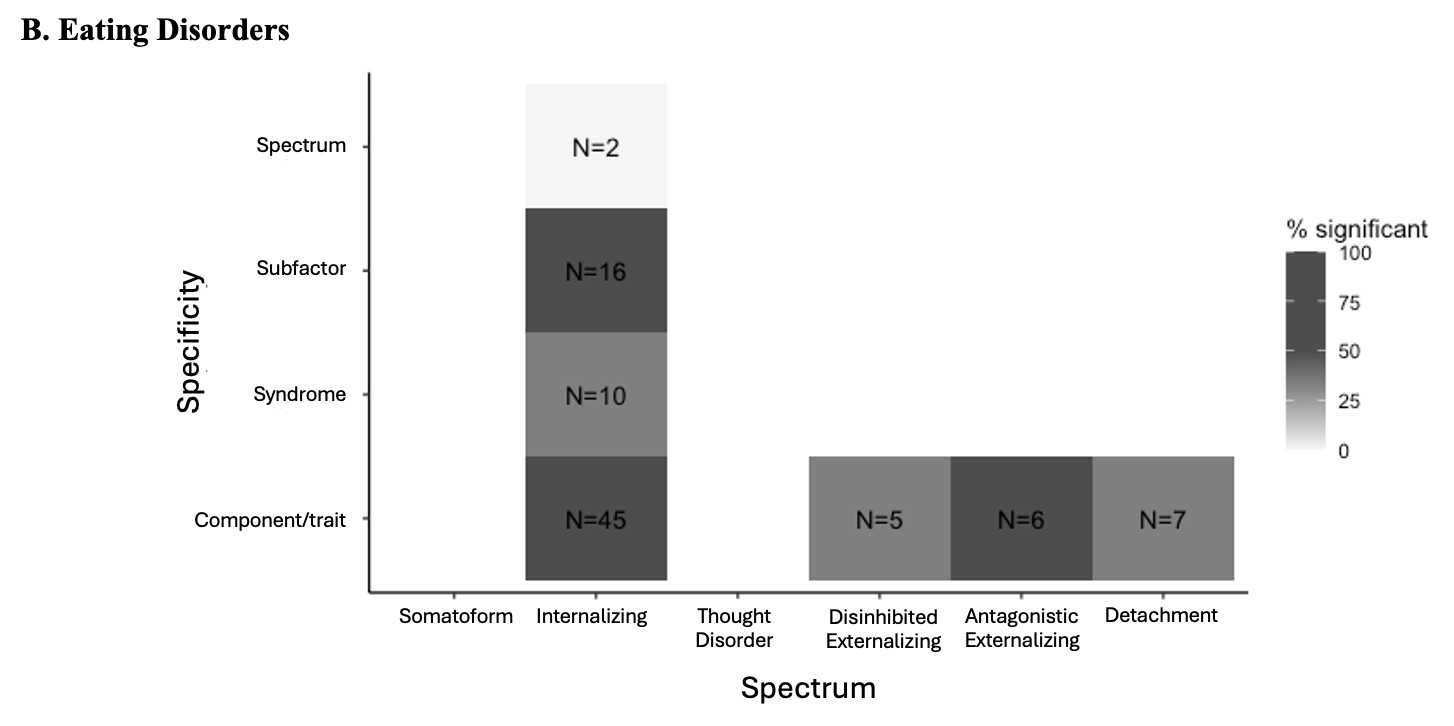


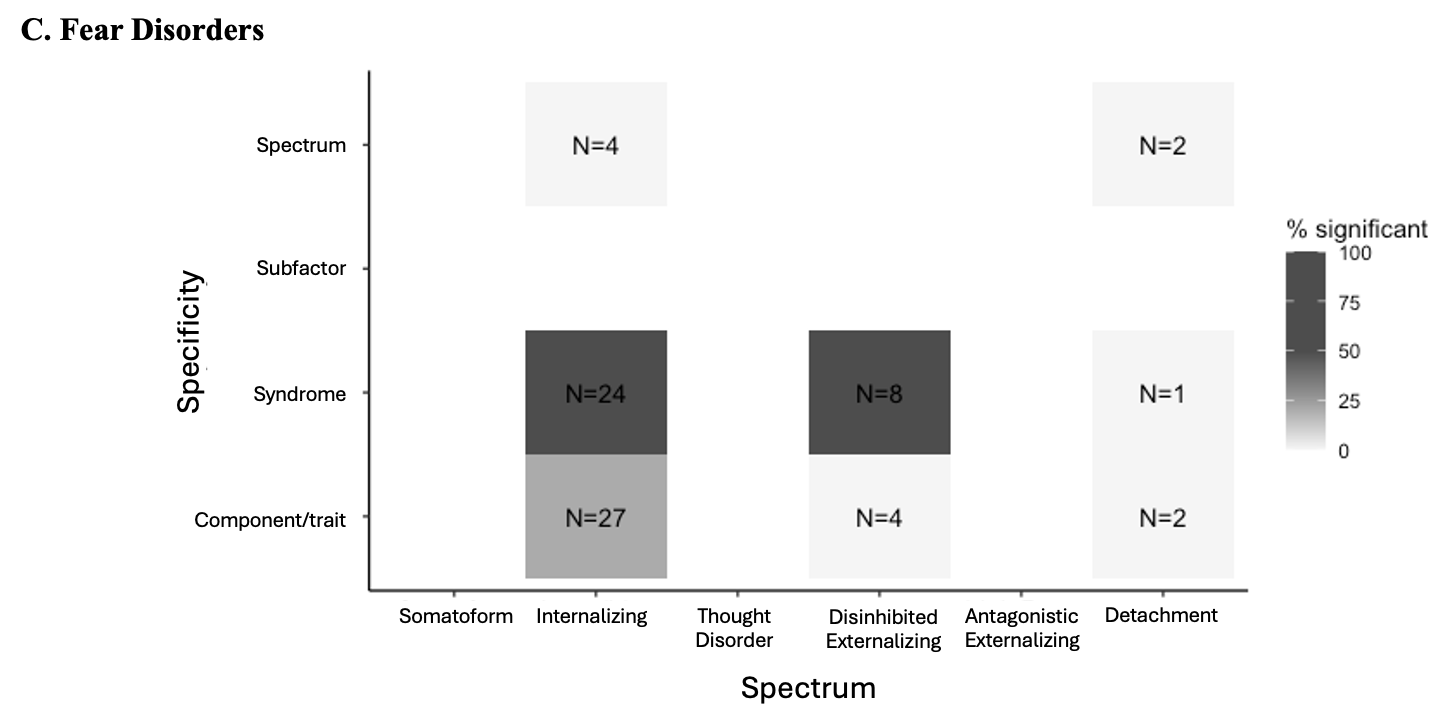


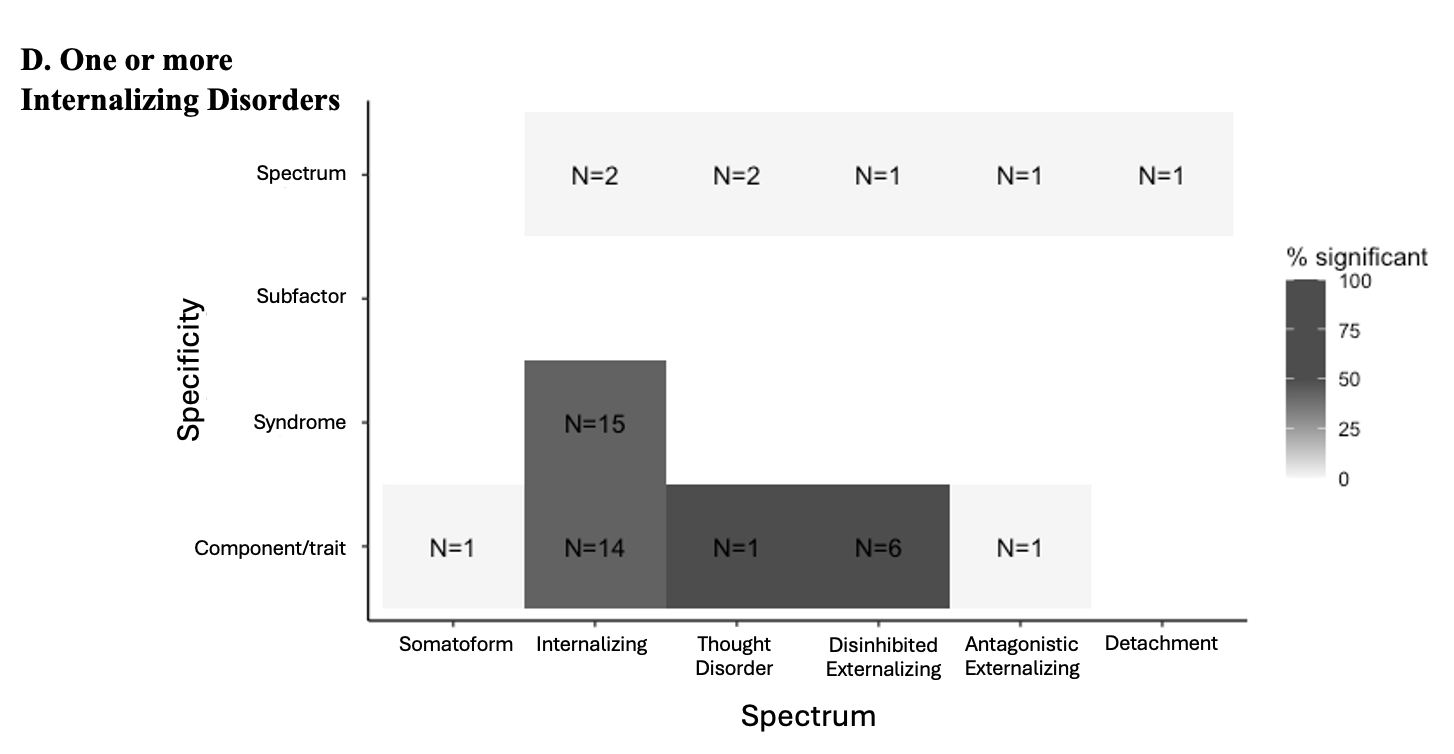


*Note*: Darker grey colored boxes correspond to higher percentage of studies with at least one significant moderating effect within a box of the heatmap. Light grey corresponds to low to 0% of studies with at least one significant effect. White space in the figure = no moderators examined in that spectrum and specificity level. N = Total number of studies testing moderators within a box of the heatmap.
